# Coancestry superposed on admixed populations yields measures of relatedness at individual-level resolution

**DOI:** 10.1371/journal.pcbi.1013848

**Published:** 2025-12-31

**Authors:** Danfeng Chen, John D. Storey

**Affiliations:** Lewis-Sigler Institute for Integrative Genomics, Princeton University, Princeton, New Jersey, United States of America; University of Auckland, NEW ZEALAND

## Abstract

The admixture model is widely applied to estimate and interpret population structure among individuals. Here we consider a “standard admixture” model that assumes the admixed populations are unrelated and also a generalized model, where the admixed populations themselves are related via coancestry (or covariance) of allele frequencies. The generalized model yields a potentially more realistic and substantially more flexible model that we call “super admixture”. This super admixture model provides a one-to-one mapping in terms of probability moments with the kinship model, the latter of which is a general model of genome-wide relatedness and structure based on identity-by-descent. We introduce a method to estimate the super admixture model that is based on method of moments, does not rely on likelihoods, is computationally efficient, and scales to massive sample sizes. We apply the method to several human data sets and show that the admixed populations are indeed substantially related, implying the proposed method captures a new and important component of evolutionary history and structure in the admixture model. We show that the fitted super admixture model estimates relatedness between all pairs of individuals at a resolution similar to the kinship model. The super admixture model therefore provides a tractable, forward-generating probabilistic model of complex structure and relatedness that should be useful in a variety of scenarios.

## Author summary

Characterizing patterns of genetic relatedness and structure among individuals is a central topic in genetics. A widely used framework for this is the admixture model, which views each individual’s genome as a mosaic derived from several antecedent populations. However, a standard admixture model assumes the antecedent populations are unrelated, an assumption that may not adequately capture the complexity of human population structure. In this work, we consider an extended model to allow the admixed antecedent populations themselves to be related to each other, a framework we call “super admixture”. We introduce algorithms for estimating the super admixture model and simulating genotypes under this model that are likelihood-free, computationally efficient, and scalable. Applying our approach to several human genetic data sets, we show that the super admixture model uncovers finer structure among the admixed populations, and thereby provides measures of relatedness at individual-level resolution.

## Introduction

Populations are structured when genotype frequencies do not follow Hardy-Weinberg proportions. This may be due to several factors, including finite population sizes, migration, and genetic drift [1,2]. Our goal here is to develop a framework and estimation method of a forward-generating probability process that captures the observed genetic structure and relatedness among a set of individuals in a population-based study.

The framework is based on covarying allele frequencies among populations [3] and individuals [4], which we will refer to as *coancestry* [3–5]. The data underlying the proposed method are single nucleotide polymorphism (SNP) genotypes measured throughout the genome on a set of individuals. The aim is to formulate and estimate a model of the underlying process that leads to individual-specific allele frequencies (IAFs), which are parameters consisting of possibly distinct allele frequencies for every individual-SNP pair. IAFs have been formulated in previous work [6,7] and they are the estimation target in several established admixture methods [8–10], a genome-wide association test for structured populations [11], and a test of structural Hardy-Weinberg equilibrium [12].

A joint probability distribution of the IAFs under a neutral model has been developed that yields covariances for all pairs of IAFs, parameterized by ancestral allele frequencies and coancestry parameters [4,5]. This model produces a one-to-one mapping with the kinship parameters from the *identity-by-descent* model [13,14], excluding close familial genetic relationships. This coancestry model therefore captures pairwise individual-level structure and relatedness equivalent to the kinship model. However, similarly to the kinship model, the coancestry parameterization is in terms of expected values, variances, and covariances of the IAFs and genotypes. It does not explicitly define a forward-generating probability model of IAFs.

Admixture models have been explored as a possible way to define such a forward-generating probability model [4,5]. The products of an admixture model are individual-specific admixture proportions and population-specific allele frequencies. The IAFs are modeled as a weighted average of these *antecedent population allele frequencies* by the *individual-specific admixture proportions*. Several methods treat the admixture proportions and antecedent population allele frequencies as unknown parameters without explicitly making any assumptions about their random distributions [8–10]. Other methods place a prior probability distribution on them for Bayesian model fitting purposes [15–17]; however, these Bayesian methods do not include these prior distributions as an inference target.

**Fig 1:**
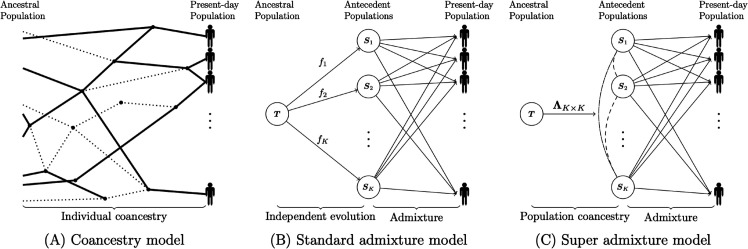
Graphical representations of the coancestry model, the standard admixture model, and the super admixture model. (A) In the coancestry model, individuals in the present-day population are connected by a complex genealogy. (B) In the standard admixture model, the arrows connecting *T* with $S_{1},\ldots ,S_{K}$ reflect that the antecedent populations evolved independently from *T*. Arrows connecting $S_{1},\ldots ,S_{K}$ with individuals in the present-day population reflect that these individuals were admixed from independent antecedent populations.(C) In the super admixture model, dashed lines connecting all pairs of antecedent populations reflect that antecedent populations have coancestry parameterized by Λ. Arrows connecting $S_{1},\ldots ,S_{K}$ with individuals in the present-day population reflect that these individuals were admixed from covarying antecedent populations.

In considering a model of random antecedent population allele frequencies, one could assume that the allele frequencies are independently generated among all antecedent populations based on a common set of parameters (e.g., independent draws from the Balding-Nichols distribution [18]). We will call this assumption the “standard admixture” model. However, this standard admixture model may be overly restrictive; rather, one could implement a coancestry model of the antecedent allele frequencies according to pairwise covariances [4,5]. We will call this model the “super admixture” model, as coancestry (or covariance) is superposed on the admixed antecedent populations. Fig 1 displays a schematic of these models.

Here, we develop a method that estimates the parameters in the super admixture model, which includes the standard admixture model as a special case. The method is based on method of moments estimation and geometric considerations, so it does not make assumptions about the probability distributions of the parameters and it does not involve costly likelihood maximization computations. Likelihood maximization is the most common approach used in fitting the admixture models [8,9,15–17], but we build from a recently proposed distribution-free moment-based method, called ALStructure, that only uses linear projections and geometric constraints on parameters to estimate the model [10]. ALStructure performs favorably to likelihood-based methods (even in achieving a high likelihood) and can be tractably scaled to massive data sets. Our proposed super admixture method complements this framework and has similar advantages.

**Fig 2:**
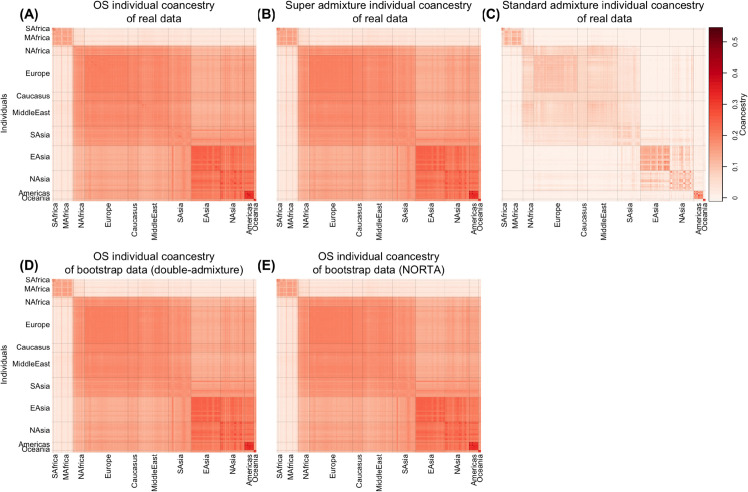
Heatmaps of individual-level coancestry estimates in the Human Origins (HO) data set. Each cell represents the estimated coancestry between a pair of individuals, with warmer colors indicating higher values. (A)–(C) show estimates from the observed genotypes using the Ochoa–Storey (OS) method, the super admixture method, and the standard admixture method, respectively. (D) shows estimates from bootstrap re-sampled genotypes using the OS method, with antecedent allele frequencies simulated under a double-admixture approach. (E) shows estimates from bootstrapped re-sampled genotypes using the OS method, with antecedent allele frequencies simulated using the NORTA approach. See Results for full details.

**Fig 3:**
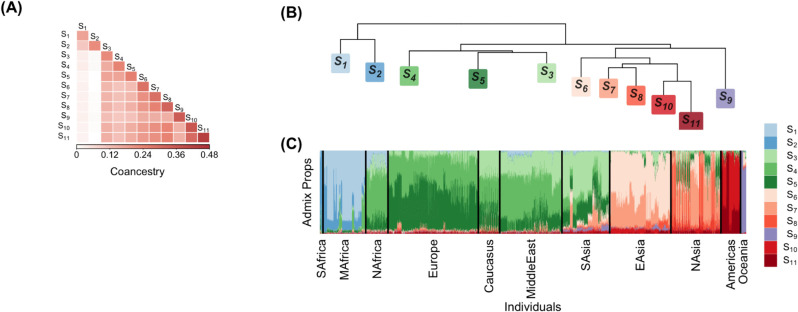
Visualization of population-level coancestry and admixture proportions in the Human Origins (HO) data set. (A) Heatmap of antecedent population coancestry estimates. (B) Dendrogram representation of the antecedent population coancestry estimates. (C) Stacked bar plot of admixture proportions. See Results for full details.

We establish super admixture through computational studies and analyses of data sets, including the human genome diversity panel (HGDP) [19,20], the 1000 genomes project (TGP) [21], the Human Origins study (HO) [22,23], and a study on individuals with Inadian ancestry (IND) [24]. We show on all of these data sets that the super admixture method is capable of capturing the same relatedness and structure as a model-free individual-level coancestry estimator [4], whereas the standard admixture model does not. We demonstrate that the framework can generate bootstrap genotypes that retain the structure seen in the human studies. For example, Fig 2 shows these results on the HO study. We show that the coancestry among antecedent populations estimated by super admixture yields new insights and visualizations of structure previously unavailable, for example, Fig 3 on the HO study. We develop and apply a statistical test demonstrating in these studies that coancestry among the admixed antecedent populations is significantly different from zero at a high level of significance.

Our proposed framework makes several contributions: (i) a distribution-free framework that can account for arbitrarily complex relationships among the admixed antecedent populations; (ii) an admixture-based estimation of individual-level pairwise coancestry at a resolution equivalent to general, model-free coancestry and kinship; (iii) a partitioning of the super admixture model into evolutionary, genealogical, and statistical sampling components; and (iv) a tractable algorithm to generate bootstrap samples of genotypes from the estimated evolutionary process.

## Methods

Here, we first introduce the data and models, and then we detail the proposed framework. We describe how the framework is used to estimate the super admixture model, generate parameters and data from the model, and perform a hypothesis test of the standard versus super admixture models.

### Coancestry

We assume that *m* SNPs are measured on *n* individuals. The genotype measurements are denoted by *x*_*ij*_ for i=1,…,m and j=1,…,n. For each SNP, one of the alleles is counted as a 0 and the other as a 1, implying that the SNP genotypes are $x_{ij}\in \{0,1,2\}$ where *x*_*ij*_ = 0 is homozygous for the 0 allele, *x*_*ij*_ = 1 is a heterozygote, and *x*_*ij*_ = 2 is homozygous for the 1 allele. We assume that $𝔼[x_{ij}|\pi_{ij}]=2\pi_{ij}$ for IAF $\pi_{ij}$. This IAF parameterization allows each individual-SNP pair to possibly have a distinct allele frequency. The classical scenario where there is one allele frequency per SNP is a special case where $\pi_{i1}=\pi_{i2}=\cdots =\pi_{in}$. The conditional expected value $𝔼[x_{ij}|\pi_{ij}]=2\pi_{ij}$ also allows for the IAFs $\pi_{ij}$ to be random parameters, which we assume here.

We utilize an existing coancestry model where the IAFs are random parameters with respect to some ancestral population *T* that is common to all *n* individuals [4,5]. (Note that other definitions of “coancestry” and “kinship” exist, but we use the models defined below and in refs. [4,5].) This is a neutral model where

$$𝔼[\pi_{ij}|T]=a_{i}$$ (1)

$$\mathbb{C}[\pi_{ij},\pi_{ik}|T]=a_{i}(1-a_{i})\theta_{jk}$$ (2)

for i=1,…,m and j,k=1,…,n. The parameter *a*_*i*_ is the ancestral allele frequency in *T* for SNP *i* and $0\leq \theta_{jk}\leq 1$ is the coancestry for individuals *j* and *k* with respect to *T*. (Note that the *a*_*i*_ and $\theta_{jk}$ parameters depend on *T* and could be different if conditioning on a different common ancestral population.) The coancestry model we utilize also makes the assumption used in previous work [4,5,7–12] that $x_{ij}|\pi_{ij}∼\text{Binomial}(2,\pi_{ij})$, where the $x_{ij}|\pi_{ij}$ are jointly independent. Under this model, it follows that


$$\mathbb{C}[x_{ij},x_{ik}|T]=\left\{ \begin{array}{cc} 2a_{i}(1-a_{i})\left(1+\theta_{jj}\right) & j=k,\\ 4a_{i}\left(1-a_{i}\right)\theta_{jk} & j\neq k. \end{array}\right.$$

A one-to-one mapping exists with the identity-by-descent kinship model (often used in GWAS methods), denoted by $\phi_{jk}$, by matching variances and covariances [4,5]. The parameters map so that

$$\theta_{jk}=\left\{ \begin{array}{cc} 2\phi_{jk}-1 & \text{if}\;j=k,\\ \phi_{jk} & \text{if}\;j\neq k. \end{array}\right.$$ (3)

When $\min_{jk}\theta_{jk}=0$, then *T* is the most recent common ancestral population [4]. The full set of parameters is denoted by the n×n symmetric matrix Θ with (*j*,*k*) entry $\theta_{jk}$.

### Admixture models

#### General admixture.

We first describe a general formulation of the admixture model. Both the standard admixture model and our proposed super admixture model can be viewed as special cases of this general formulation. There are *K* populations $S_{1},S_{2},\ldots ,S_{K}$ descended from *T* that precede the present-day population, which we refer to as “antecedent populations”. While *T* has allele frequencies $a_{1},a_{2},\ldots ,a_{m}$, antecedent population *S*_*u*_ has allele frequencies $p_{1u},p_{2u},\ldots ,p_{mu}$ for u=1,2,…,K. The allele frequencies {*p*_*iu*_} are random parameters from a distribution parameterized by {*a*_*i*_} plus other possible parameters that characterize the evolutionary process from *T* to *S*_*u*_.

For each individual *j*, there is a genealogical process from population *T* to the present-day population. This is captured by a random *K*-vector $q_{1j},q_{2j},\ldots ,q_{Kj}$ of admixture proportions, where $0\leq q_{uj}\leq 1$ and $\sum_{u=1}^{K}q_{uj}=1$. The parameter *q*_*uj*_ is the proportion of the individual *j* randomly descended from *S*_*u*_. Therefore, the IAFs are such that

$$\pi_{ij}=\sum_{u=1}^{K}p_{iu}q_{uj}.$$ (4)

We collect the antecedent population allele frequencies into the m×K matrix P and the admixture proportions into the K×n matrix Q; it follows that


Π=PQ,

where Π is an m×n matrix with (*i*,*j*) entry $\pi_{ij}$.

#### Standard admixture.

We define the standard admixture model to be the case where the antecedent allele frequencies are independently distributed. Specifically, in this model *p*_*iu*_ is a random parameter with mean *a*_*i*_ and variance $a_{i}(1\;-\;a_{i})f_{u}$. The standard admixture model is defined as follows for i=1,2,…,m and u=1,2,…,K.


$$\text{Standard Admixture:}\;p_{i1},p_{i2},\ldots ,p_{iK}\text{ are jointly }\mathrm{independent}\;𝔼[p_{iu}|T]=a_{i}\;𝕍[p_{iu}|T]=a_{i}(1-a_{i})f_{u}$$

Under this parameterization, *a*_*i*_ is the ancestral allele frequency in *T* and *f*_*u*_ is the inbreeding coefficient or $F_{\text{ST}}$ of antecedent population *S*_*u*_ with respect to *T*. Since the {*p*_*iu*_} are jointly independent, there is no coancestry among antecedent populations and there is no dependence among loci.

One well-known distribution that could be utilized here is the Balding-Nichols (BN) distribution [18] with parameters *a*_*i*_ and *f*_*u*_:

$$p_{iu}∼\text{Beta}\left(\frac{1-f_{u}}{f_{u}}a_{i},\frac{1-f_{u}}{f_{u}}(1-a_{i})\right).$$ (5)

We will write this re-parameterized Beta distribution as $\text{BN}(a_{i},f_{u})$. This achieves the expected value and variance of the standard admixture definition. The Balding-Nichols distribution is often used to generate allele frequencies for a set of populations to achieve desired expected allele frequencies and $F_{\text{ST}}$ values. This distribution has been discussed as useful for generating antecedent allele frequencies in the standard admixture model [4–7,25].

#### Super admixture.

The super admixture model extends the standard admixture model in that it includes a covariance among antecedent population allele frequencies, which we refer to as population-level coancestry. While we denoted individual-level coancestry by $\theta_{jk}$, we will denote population-level coancestry by $\lambda_{uv}$ for u,v=1,2,…,K where $0\leq \lambda_{uv}\leq 1$. We collect these values into the K×K symmetric coancestry matrix Λ. The super admixture model is defined as follows for i=1,2,…,m and u,v=1,2,…,K.

$$\text{Super Admixture:}\;p_{i1},p_{i2},\ldots ,p_{iK}\text{ are jointly }\mathrm{dependent}\;𝔼[p_{iu}|T]=a_{i}\;𝕍[p_{iu}|T]=a_{i}(1-a_{i})\lambda_{uu}\;\mathbb{C}[p_{iu},p_{iv}|T]=a_{i}(1-a_{i})\lambda_{uv}$$ (6)

In this model we assume that allele frequencies between loci are independent, so the random *K*-vectors $\left(p_{h1},p_{h2},\ldots ,p_{hK}\right)$ and $\left(p_{i1},p_{i2},\ldots ,p_{iK}\right)$ are independent for h⧸=i. Thus, a potential generalization of the super admixture model is to include dependence among loci. Otherwise, the super admixture model is general in that it allows for the full range of coancestry values among antecedent populations.

#### Forward-generating probability process.

We now describe the super admixture model as a forward-generating probability process. Suppose that the admixture proportions Q are drawn from some probability distribution 𝒬. Then, for i=1,2,…,m and j=1,2,…,n:


$$\left(p_{i1},p_{i2},\ldots ,p_{iK})∼(a,\Lambda )\;(q_{1j},q_{2j},\ldots ,q_{Kj})∼𝒬\;\pi_{ij}=\sum_{u=1}^{K}p_{iu}q_{uj}\;x_{ij}|\pi_{ij}∼\text{Binomial}(2,\pi_{ij}\right)$$

The joint probability of all random quantities can be factored as follows:


ℙ(X,Q,P|T,𝒬)=ℙ(P|T)ℙ(Q|𝒬)ℙ(X|P,Q).

One interpretation of this is that ℙ(P|T) represents evolutionary sampling, ℙ(Q|𝒬) represents genealogical sampling, and ℙ(X|P,Q) represents statistical sampling.

#### Individual-level coancestry in the admixture models.

Recall that in the coancestry model, the covariance of two IAFs for a given SNP is $\mathbb{C}[\pi_{ij},\pi_{ik}|T]=a_{i}(1-a_{i})\theta_{jk}$, shown in Eq (2). Conditioning on the admixture proportions Q, which are ancillary to antecedent allele frequencies, this covariance under the super admixture model is, for j,k=1,2,…,n,

$$\mathbb{C}\left[\pi_{ij},\pi_{ik}|Q,T\right]=\mathbb{C}\left[\sum_{u=1}^{K}p_{iu}q_{uj},\sum_{v=1}^{K}p_{iv}q_{vk}|Q,T\right]\;=\sum_{u=1}^{K}\sum_{v=1}^{K}q_{uj}q_{vk}\mathbb{C}\left[p_{iu},p_{iv}|T\right]\;=a_{i}(1-a_{i})\sum_{u=1}^{K}\sum_{v=1}^{K}q_{uj}q_{vk}\lambda_{uv}.$$ (7)

By setting the covariance from Eq (2) equal to Eq (7), it follows that under the super admixture model the individual-level coancestry is the following.

$$\text{Super Admixture Individual-level Coancestry:}\;\theta_{jk}=\sum_{u=1}^{K}\sum_{v=1}^{K}q_{uj}q_{vk}\lambda_{uv}$$ (8)

In the standard admixture model, $𝕍[p_{iu}|T]=a_{i}(1-a_{i})f_{u}$, whereas in the super admixture model $𝕍[p_{iu}|T]=a_{i}(1-a_{i})\lambda_{uu}$. If we set $f_{u}=\lambda_{uu}$, the difference between the standard and super admixture models is therefore that in the standard model, $\lambda_{uv}=0$ for u⧸=v. To work with a single notation, we will therefore write $\lambda_{uu}$ in place of *f*_*u*_ for the standard admixture model. The coancestry in this model is as follows.

$$\text{Standard Admixture Individual-level Coancestry:}\;\theta_{jk}=\sum_{u=1}^{K}q_{uj}q_{uk}\lambda_{uu}\;\lambda_{uv}=0\text{ for }u\text{⧸}=v$$ (9)

Considering all pairs of individuals simultaneously, the individual-level coancestry matrix Θ can be written in terms of the antecedent population-level coancestry Λ and the admixture proportions Q as


$$\Theta =Q^{\prime }\Lambda Q,$$

which is an important relationship we utilize to estimate Λ.

### Estimating coancestry among antecedent populations

Here, we propose a method to estimate the antecedent population-level coancestry Λ under the super admixture model, with the standard admixture model estimate as a special case. The rationale is to leverage the relationship, $\Theta =Q^{\prime }\Lambda Q$. Given values for Θ and Q, we identify values of Λ that make $Q^{\prime }\Lambda Q$ close to Θ, while obeying the geometic constraints of Λ (i.e., $0\leq \lambda_{uv}\leq 1$ and $\lambda_{uv}=\lambda_{vu}$).

Given values for Θ and Q, we formulate the problem of estimating the antecedent population-level coancestry Λ under the super admixture model as follows.

**Problem 1.**


$$\min_{\Lambda \in {\mathbb{R}}^{K\times K}}\|\Theta -Q^{\prime }\Lambda Q{\|}_{F}^{2}\;\text{subject to: }0\leq \lambda_{uv}\leq 1\text{ and }\lambda_{uv}=\lambda_{vu}\;\text{for }u,v=1,\ldots ,K$$

where $\|\cdot {\|}_{F}$ represents the Frobenius norm (Appendix A, S1 Text). We utilize the proximal forward-backward (PFB) method [26] to solve this optimization problem, resulting in Algorithm 1 for solving Problem 1. Every sequence of $(\Lambda_{t}{)}_{t\in \mathbb{N}}$ generated from this algorithm is guaranteed to converge to a solution of the corresponding problem. The PFB method and how to employ it in our setting are detailed in Appendix D in S1 Text. The performance of Algorithm 1 is demonstrated in Appendix J in S1 Text.

**Algorithm 1. Estimating Λ for the super admixture model given Θ and Q.**



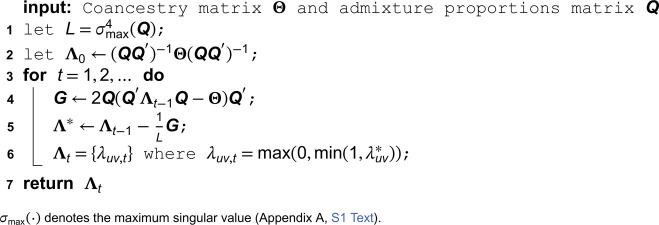



Note that since the optimization criterion for fitting Λ is driven by relatedness averaged over the genome as quantified by Θ, there may be localized signals that show interesting admixture events. To this end, one could pursue an optimization on a more localized basis. Also, note that rotations of Q and Λ can yield the same product $Q^{\prime }\Lambda Q$, so it is important to not over-interpret Q and Λ, which is true of admixture models in general [10].

To estimate all components of the super admixture model, one needs estimates of the n×n individual-level coancestry matrix Θ, the K×K antecedent population-level coancestry matrix Λ, the m×K matrix of antecedent population allele frequencies P, and the K×n matrix of admixture proportions Q. There exists a wide range of methods for estimating P and Q [8–10,15–17]. Here, we utilize the ALStructure method [10], which is formulated under the general admixture model with no assumptions about the structure of Λ. Therefore, ALStructure applies equally to both the super admixture and the standard admixture settings. This is also the case for some other methods, such as ADMIXTURE [9]. The method proceeds by deriving a linear basis for Q from X that has theoretical guarantees to span the true basis as the number of SNPs *m* grows large. A projection-based estimate $\hat{\Pi }$ of the IAFs is also formed. The quantity $\|\hat{\Pi }\;-\;QP{\|}_{F}$ is then algorithmically minimized through geometrically constrained alternating least squares to yield estimates $\hat{Q}$ and $\hat{P}$.

We utilize the structural Hardy-Weinberg (sHWE) test [12] for determining the number of antecedent populations *K*, as outlined in that work. The approach is to consider a range of *K* values to test the assumption that $x_{ij}|\pi_{ij}∼\text{Binomial}(2,\pi_{ij})$ based on the estimates ${\hat{\pi }}_{ij}$ and a goodness-of-fit statistic with a parametric bootstrap null distribution; *K* is then parsimoniously chosen to satisfy this modeling assumption from a genome-wide perspective. A method of moments estimator of Θ was derived in [4], where it was shown to have favorable properties and is consistent for the true values under certain assumptions. We denote this Ochoa-Storey (OS) estimate by ${\hat{\Theta }}^{\text{OS}}$ and review its details in Appendix C in S1 Text. If one has alternative ways to estimate Θ and Q, and to determine *K*, then those can be used within our framework as well.

Note that one can further calculate a corresponding estimate for individual-level coancestry by


$${\hat{\Theta }}^{\text{sup}}={\hat{Q}}^{\prime }{\hat{\Lambda }}^{\text{sup}}\hat{Q},$$

which can be compared to ${\hat{\Theta }}^{\text{OS}}$ in order to aid in model fit assessment.

We can estimate Λ under the standard admixture model by modifying the constraints in Problem 1. This leads to the Algorithm B described in Appendix D in S1 Text. Algorithm 2 can then be used to form the estimate ${\hat{\Lambda }}^{\text{std}}$ under the standard admixture model with Algorithm 1 replaced by Algorithm B in Line 4. The corresponding estimate for individual-level coancestry can be calculated as ${\hat{\Theta }}^{\text{std}}={\hat{Q}}^{\prime }{\hat{\Lambda }}^{\text{std}}\hat{Q}$. The performance of Algorithm B is also demonstrated in Appendix J in S1 Text.

**Algorithm 2. Estimating Λ for the super admixture model given X.**

**input:** Genotype matrix X

**1** calculate the OS estimate of individual-level coancestry ${\hat{\Theta }}^{\text{OS}}$;

**2** choose *K* from the structural Hardy-Weinberg (sHWE) goodness of fit procedure;

**3** calculate the estimate $\hat{Q}$ for *K* via the ALStructure method;

**4** calculate the estimate ${\hat{\Lambda }}^{\text{sup}}$ by applying Algorithm 1 with inputs ${\hat{\Theta }}^{\text{OS}}$ and $\hat{Q}$ ;

**5 return** ${\hat{\Lambda }}^{\text{sup}}$

### Simulating antecedent population allele frequencies

We now introduce a method to generate antecedent population allele frequencies with given coancestry Λ. We note above in Eq (5) that for the standard admixture model, one way to generate allele frequencies $p_{i1},p_{i2},\ldots ,p_{iK}$ is via independent realizations from the Balding-Nichols (BN) distribution: $p_{iu}∼\text{BN}(a_{i},\lambda_{uu})$ for u=1,2,…,K. As there is no default approach to extending this to the super admixture case, we propose a method here called “double-admixture”. The main idea of the method is that we form two layers of allele frequencies: the first layer is composed of independent draws from the BN distribution, and the second layer mixes these to create $p_{i1},p_{i2},\ldots ,p_{iK}$ with coancestry Λ.

Let *S* be the number of components that will be mixed, W be the S×K matrix of mixture proportions, and Γ an S×S diagonal matrix. The entries of W are *w*_*su*_ where $0\leq w_{su}\leq 1$ and $\sum_{s=1}^{S}w_{su}=1$ for u=1,2,…,K. The diagonal values of Γ are represented by $\gamma_{s}$ where $0\leq \gamma_{s}\leq 1$, and all other values are 0. Suppose that for i=1,…,m we generate


$$z_{is}∼\text{BN}(a_{i},\gamma_{s})$$

independently for s=1,…,S, and we then set


$$p_{iu}=\sum_{s=1}^{S}z_{is}w_{su}$$

for u=1,…,K. It can be verified that


$$𝔼[p_{iu}]=a_{i}\;u=1,\ldots ,K$$

$$\mathbb{C}[p_{iu},p_{iv}]=a_{i}(1-a_{i})\sum_{s=1}^{S}w_{su}w_{sv}\gamma_{s}$$

for u,v=1,2,…,K. By matching these equations with Eq (6), one can see that if

$$\lambda_{uv}=\sum_{s=1}^{S}w_{su}w_{sv}\gamma_{s}$$ (10)

then $p_{i1},p_{i2},\ldots ,p_{iK}$ has coancestry Λ as desired. In matrix terms, Eq (10) is equivalent to

$$\Lambda =W^{\prime }\Gamma W.$$ (11)

Therefore, the double-admixture method is based on the following optimization problem.

**Problem 2.**


$$\min_{W,\Gamma }\|\Lambda -W^{\prime }\Gamma W{\|}_{F}^{2}$$

$$\text{subject to: }0\leq w_{su}\leq 1,\sum_{s=1}^{S}w_{su}=1$$

$$\epsilon \leq \gamma_{s}\leq 1-\epsilon \text{ for small }\epsilon >0$$

for u=1,2,…,K;s=1,2,…,S

We adopt the proximal alternating linearized minimization (PALM) method [27] to solve Problem 2, resulting in Algorithm 3 for calculating the parameters in the double-admixture method. Every sequence $(W_{t},\Gamma_{t}{)}_{t\in \mathbb{N}}$ generated from Algorithm 3 is guaranteed to converge to a critical point. Integrating Algorithm 3 with the generative steps for *p*_*iu*_ described above, Algorithm 4 simulates antecedent population allele frequencies with the desired coancestry. We note that the parameter *S* should be chosen to ensure that $\Lambda \approx W^{\prime }\Gamma W$ while avoiding unnecessarily large values, which may increase computational time without improving accuracy. A practical guideline is to plot $\|\Lambda -W^{\prime }\Gamma W{\|}_{F}$ against *S* and identify a value of *S* where the curve plateaus; empirically, setting *S* = 2*K* works well in the examples considered here. In Appendix E in S1 Text, the PALM method is briefly introduced and the convergence of Algorithm 3 is proved.

**Algorithm 3. Calculating W and Γ in the double-admixture method.**



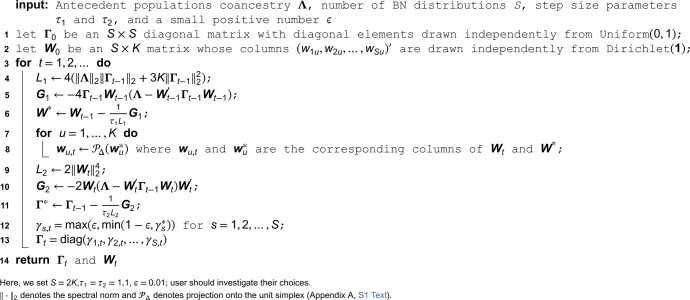



**Algorithm 4. The double-admixture algorithm for simulating P.**



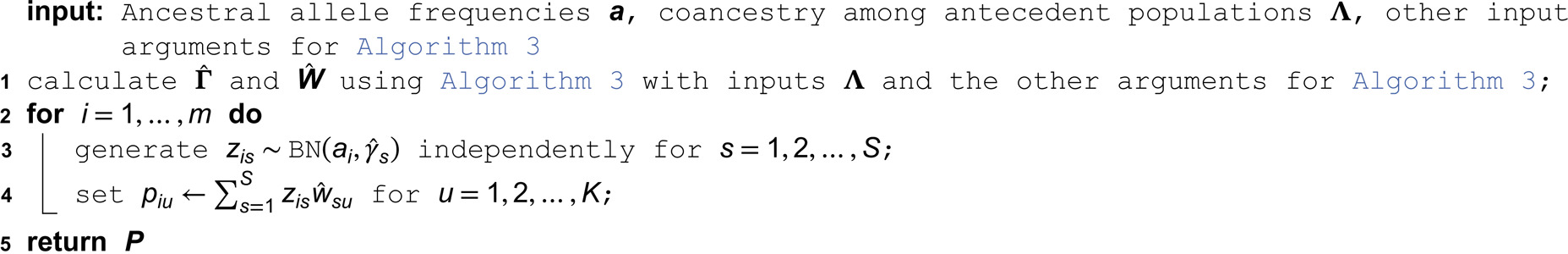



One possible drawback of the double-admixture method is that the approach relies on the existence of W and Γ so that $\Lambda =W^{\prime }\Gamma W$. We do not currently have a theoretical guarantee for such W and Γ (although one may exist since *S* can be made large). Therefore, we provide a complementary method in Appendix F in S1 Text, the NORmal To Anything (NORTA) approach [28], serving as a tool for simulating P when the double-admixture method is not applicable. It should be noted that the double-admixture method solves the optimization one time for the entire process so that its running time is independent of the number of loci *m*. In contrast, the NORTA method has to solve K×(K−1)/2 root-finding problems per locus and therefore has a complexity of $𝒪(K^{2}m)$, rendering it significantly more time consuming. The performances of the double-admixture and NORTA methods are demonstrated in Appendix K in S1 Text.

Note that if we set Γ=Λ for a diagonal standard admixture Λ and $W=I_{K}$ (where $I_{K}$ is the K×K identity matrix), then the double-admixture method reduces to the BN sampling from Eq (5), which produces valid antecedent population frequencies for the standard admixture model. From this observation, the double-admixture method can be viewed as a generalization of BN sampling.

### Generating bootstrap data sets from realistic population structures

By utilizing the double-admixture method, we introduce Algorithm 5 to generate genotypes under the super admixture model. When the true parameters (a,Λ,Q) are available, the algorithm can be used for direct simulation. We verify in Appendix L of S1 Text that the generated genotypes satisfy the moment constraints imposed by the super admixture model. When these parameters are not available, the same procedure can be applied in a semi-parametric bootstrap framework by first estimating them from observed genotype data. Specifically, $\hat{Q}$ can be obtained with ALStructure, $\hat{\Lambda }$ with the proposed super admixture estimator, and $\hat{a}$ with the vector of observed allele frequencies from each SNP. In this setting, the algorithm yields bootstrap replicates $X^{*}$ to recapitulate the structure and relatedness of the observed data X. Importantly, the bootstrap process does not merely resample genotypes from a fixed matrix of estimated individual allele frequencies (IAFs). Rather, the antecedent population allele frequencies are resampled, also leading to resampled IAFs, so both evolutionary and statistical resampling occur.

**Algorithm 5. Generating genotypes from the super admixture model.**



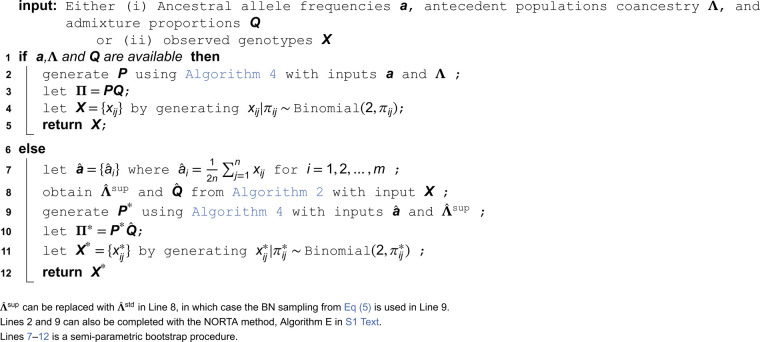



### Significance test of coancestry among antecedent populations

Here, we develop a hypothesis test of the standard admixture model (null) versus the super admixture model (alternative). We show below that on real data sets the test results are highly significant against the null in favor of the alternative. In terms of model parameters, the test is defined as follows:


$$H_{0}:\max\left(\{\lambda_{uv}{\}}_{u\neq v}\right)=0\text{ (standard admixture model)}$$

$$H_{1}:\max\left(\{\lambda_{uv}{\}}_{u\neq v}\right)>0\text{ (super admixture model)}$$

A straightforward test-statistic is $U=\|{\hat{\Lambda }}^{\text{sup}}\;-\;{\hat{\Lambda }}^{\text{std}}{\|}_{F}$. The larger *U* is, the more evidence there is against the null hypothesis in favor of the alternative hypothesis. In order to calculate a *p*-value for this test-statistic, we need to know the distribution of *U* when the null hypothesis is true. To this end, we adapt the bootstrap method of Algorithm 5, leading to Algorithm 6.

**Algorithm 6. Hypothesis test of no coancestry among antecedent populations.**



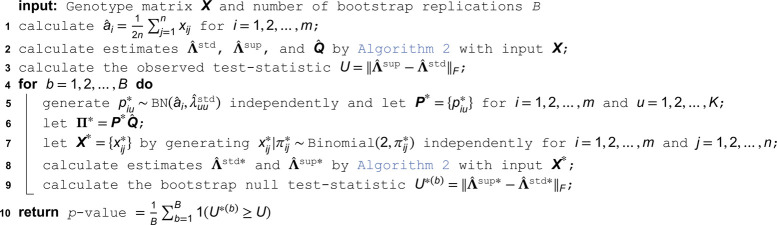



To evaluate the validity of the proposed test, we performed this hypothesis testing on various simulation designs (Appendices H and I, S1 Text). Our simulations show that the test produces valid *p*-values, which are conservative (Fig D in Appendix M, S1 Text), meaning the test has a maximum type I error rate less than or equal to the nominal level of the test. On real data sets analyzed below, these *p*-values are small, so the conservative behavior that we observe in simulations does not appear to be relevant for populations with nontrivial levels of structure.

## Results

We applied the super admixture framework to four published studies: the human genome diversity panel (HGDP) [20], the 1000 genomes project (TGP) [21], the Human Origins study (HO) [22,23], and a study on individuals with Indian ancestry (IND) [24]. Within the TGP study, we also analyzed a subset of admixed populations with American ancestry, denoted by AMR. While HGDP, TGP, and HO are sampled from ancestries throughout the world, the IND and AMR data sets are regionally sampled. This yielded five data sets that collectively represent a range of population structures and study designs. Discussions of the results on HO, AMR, and IND are in the main text, while HGDP and TGP are respectively in Appendices O and P, S1 Text.

### Calculations

We processed each data set and performed quality control (Appendix N, S1 Text). Algorithm 2 was then applied to estimate antecedent population coancestry under the super admixture model (${\hat{\Lambda }}^{\text{sup}}$) and Algorithm B under the standard admixture model (${\hat{\Lambda }}^{\text{std}}$), along with their corresponding individual-level coancestry matrices (${\hat{\Theta }}^{\text{sup}}$ and ${\hat{\Theta }}^{\text{std}}$). As a part of these algorithms, the number of antecedent populations *K* was determined using the structural Hardy–Weinberg method [12] (Appendix S, S1 Text), yielding values from *K* = 11 for HO to *K* = 3 for AMR, consistent with earlier studies [10,12,29]. Admixture proportion matrices $\hat{Q}$ were estimated with ALStructure [10].

To assess accuracy, we compared ${\hat{\Theta }}^{\text{sup}}$ and ${\hat{\Theta }}^{\text{std}}$ to the Ochoa–Storey (OS) estimate ${\hat{\Theta }}^{\text{OS}}$ [4], a consistent estimator under general population structures that makes no assumptions about IAF or coancestry distributions. As such, ${\hat{\Theta }}^{\text{OS}}$ provides a natural benchmark (Appendix C, S1 Text), allowing us to observe if the super admixture or standard admixture models lose information about individual-level coancestry relative to OS. Table A in S1 Text shows that Frobenius distances between ${\hat{\Theta }}^{\text{sup}}$ and ${\hat{\Theta }}^{\text{OS}}$ are about 10–40 times smaller than those between ${\hat{\Theta }}^{\text{std}}$ and ${\hat{\Theta }}^{\text{OS}}$. The difference between ${\hat{\Theta }}^{\text{sup}}$ and ${\hat{\Theta }}^{\text{OS}}$ is arguably practically irrelevant, meaning that ${\hat{\Theta }}^{\text{sup}}$ achieves the resolution of ${\hat{\Theta }}^{\text{OS}}$ for practical purposes.

We further tested the super admixture model against the standard admixture model using Algorithm 6 with *B* = 1000 bootstrap iterations. In all five data sets, none of the bootstrap null statistics exceeded the observed statistic, giving *p* < 0.001 (Fig I, S1 Text). To generate bootstrap replicates $X^{*}$, we applied Algorithm 5 to each observed X, using both the double-admixture method (Algorithm 4) and the NORTA method (Algorithm E, S1 Text). For each replicate, we computed the OS estimate ${\hat{\Theta }}^{\text{OS}*}$ of individual-level coancestry.

### Visualizing results

We visualized the results in two complementary ways. First, we constructed heatmaps of individual-level coancestry estimates ${\hat{\Theta }}^{\text{OS}}$, ${\hat{\Theta }}^{\text{sup}}$, and ${\hat{\Theta }}^{\text{std}}$, along with bootstrap-based estimates ${\hat{\Theta }}^{\text{OS}*}$ obtained using both the double-admixture and NORTA methods. Across all data sets, ${\hat{\Theta }}^{\text{OS}}$ and ${\hat{\Theta }}^{\text{sup}}$ were nearly indistinguishable, consistent with their close agreement (Table A, S1 Text), while ${\hat{\Theta }}^{\text{std}}$ differed substantially, indicating the standard admixture model is not sufficient for these data sets.

Second, we extended the standard stacked bar plots of estimated admixture proportions $\hat{Q}$ by incorporating the covariance structure among the antecedent populations, as captured by the super admixture coancestry matrix ${\hat{\Lambda }}^{\text{sup}}$. This matrix encodes additional information beyond $\hat{Q}$, revealing how populations themselves are related. To visualize it, we displayed both a heatmap of ${\hat{\Lambda }}^{\text{sup}}$ and a dendrogram from a distance matrix derived from ${\hat{\Lambda }}^{\text{sup}}$ (Appendix G, S1 Text; see also [30]). The dendrogram, built from the standard agglomerative clustering algorithm, provides an intuitive summary of relationships among populations, but it would only reflect true phylogeny if the assumptions of the clustering algorithm are satisfied, which should be evaluated on a case-by-case basis. Taken together, these plots connect the relationships among populations with the admixture proportions of individuals in a way that the standard model cannot.

### Human Origins (HO) study

The Human Origins data sets (HO) consists of 2124 individuals from 170 sub-subpopulations grouped into 11 subpopulations. We observed the estimated individual-level coancestry agrees with current knowledge of early human migrations [31–34]. In Fig 2, we observed the first major split between Sub-Saharan Africa and North Africa. This split reflects the divergence between Sub-Saharan Africans and the rest of human populations resulting from an out-of-Africa migration around 50-60 kya. Another split occurred between South Asia and East Asia, revealing the separation between West Eurasians and East Asians around 40-45 kya. Among the East Asia clade, we identified that the Oceanians have highest coancestry within and lowest coancestry between other subpopulations, consistent with the theory that Oceanians split earliest from the rest of East Asians.

The coancestry among antecedent populations is also compatible with early human dispersals (Fig 3). Specifically, in the dendrogram plot of the antecedent population coancestry (Fig 3B), we noted that the first branch split individuals from Sub-Saharan Africa represented by the antecedent populations *S*_1_ and *S*_2_ from individuals outside of Sub-Saharan Africa represented by the other antecedent populations. Individuals outside of Sub-Saharan Africa further branched off into two lineages: the West Eurasians represented by antecedent populations *S*_3_, *S*_4_ and *S*_5_, and the East Asians represented by antecedent populations *S*_6_ - *S*_11_. Then the Oceanians represented by the antecedent population *S*_9_ split off from the majority of East Asian ancestry, while the latter further diverged into present-day Asians (antecedent populations *S*_6_, *S*_7_, *S*_8_) and present-day Americans (antecedent populations *S*_10_ and *S*_11_).

### Admixed individuals (AMR) from the 1000 Genomes Project (TGP)

**Fig 4:**
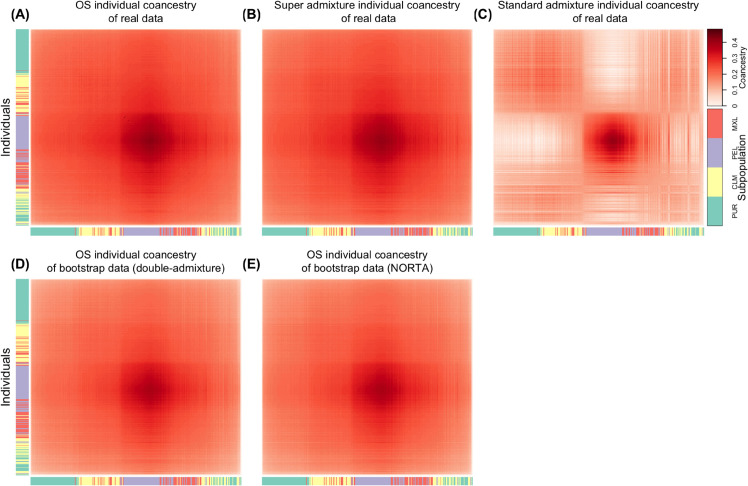
Heatmaps of individual-level coancestry estimates in admixed individuals (AMR) from the 1000 Genomes Project. Each cell represents the estimated coancestry between a pair of individuals, with warmer colors indicating higher values. (A)–(C) show estimates from the observed genotypes using the Ochoa–Storey (OS) method, the super admixture method, and the standard admixture method, respectively. (D) shows estimates from bootstrap re-sampled genotypes using the OS method, with antecedent allele frequencies simulated under a double-admixture approach. (E) shows estimates from bootstrapped re-sampled genotypes using the OS method, with antecedent allele frequencies simulated using the NORTA approach.

The AMR subset of TGP has 353 individuals from four regions (Mexican-American (MXL): 65, Puerto Rican (PUR): 104, Colombian (CLM): 97, Peruvian (PEL): 87). The individual-level coancestry plot (Fig 4) revealed that this data set does not have a discrete population structure. Instead, the coancestry changes smoothly over individuals, indicating wide-ranging historical admixture events. This is consistent with the AMR population descending from European, Native American, and Sub-Saharan African ancestries during the post-Columbian era [35,36].

**Fig 5:**
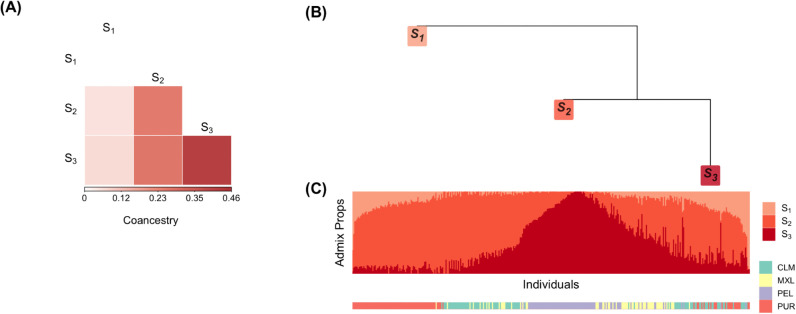
Visualization of population-level coancestry and admixture proportions in admixed individuals (AMR) from the 1000 Genomes Project. (A) Heatmap of antecedent population coancestry estimates. (B) Dendrogram representation of the antecedent population coancestry estimates. (C) Stacked bar plot of admixture proportions.

In the analysis of the coancestry among antecedent populations (Fig 5), we identified three major sources of ancestry: Sub-Saharan African ancestry represented by the antecedent population *S*_1_, West Eurasian ancestry represented by the antecedent population *S*_2_, and Native American ancestry represented by the antecedent population *S*_3_. The first split occurred between Sub-Saharan Africans (*S*_1_) and individuals outside of Sub-Saharan Africa (*S*_2_ and *S*_3_), and the second split between the West Eurasians (*S*_2_) and the Native Americans (*S*_3_). We also noted that the Puerto Ricans contain the highest amount of Sub-Saharan African ancestry; the Peruvians have the highest proportion of Native American ancestry; the Colombians and the Mexican-Americans display extensive variation in in their admixture proportions of European and Native American ancestry. Our observations were confirmed by previous analyses of AMR populations [29,35,36].

### Indian (IND) study

**Fig 6:**
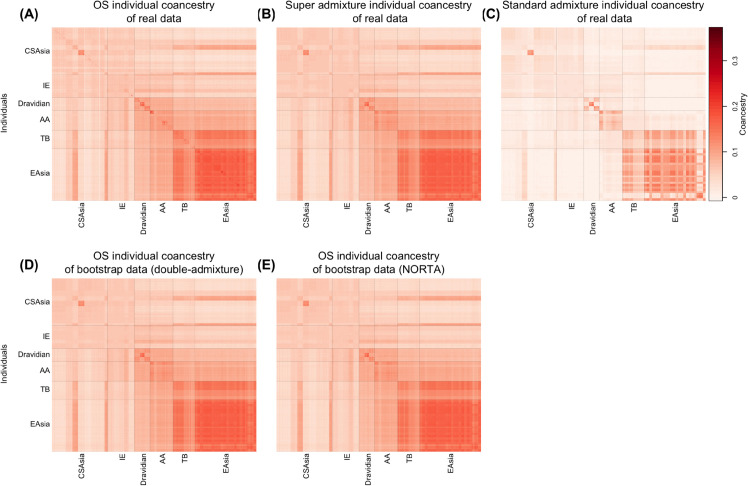
Heatmaps of individual-level coancestry estimates in the merged data set of mainland Indians from IND, and Central/South Asians and East Asians from HGDP. Each cell represents the estimated coancestry between a pair of individuals, with warmer colors indicating higher values. (A)–(C) show estimates from the observed genotypes using the Ochoa–Storey (OS) method, the super admixture method, and the standard admixture method, respectively. (D) shows estimates from bootstrap re-sampled genotypes using the OS method, with antecedent allele frequencies simulated under a double-admixture approach. (E) shows estimates from bootstrapped re-sampled genotypes using the OS method, with antecedent allele frequencies simulated using the NORTA approach.

We combined the mainland Indians from the IND study with the Central/South Asia and the East Asia populations from the HGDP to study the relationship between present-day Indians and other populations in Asia. Our merged data set consists of 298 mainland Indians from four linguistic groups (Indo-European (IE): 92, Dravidian: 53, Austro-Asiatic (AA): 79, Tibeto-Burman (TB): 74), together with 190 Central/South Asians and 210 East Asians from HGDP. Previous analyses of South Asian populations have shown that the Indo-European speakers show a considerable amount of the Western Eurasian relatedness and are ancestrally close to Central Asians. The Austro-Asiatic speakers and the Tibeto-Burman speakers were mixed from East Asian ancestry. The Tibeto-Burman speakers generally have significant genomic proportions derived from East Asian ancestry so that some Tibeto-Burman speakers can be difficult to distinguish from East Asian populations based on genome-wide measures of relatedness. Consistent with these findings [24,37,38], we observe a split between Indo-European speakers and the rest of mainland Indians in the heatmap of individual-level coancestry (Fig 6). The Indo-European speakers and the Central/South Asians of HGDP have relatively similar levels of coancestry. The second split occurred between the Austro-Asiatic speakers and the Tibeto-Burman speakers. The Tibeto-Burman speakers and East Asians of HGDP have relatively similar levels of coancestry.

**Fig 7:**
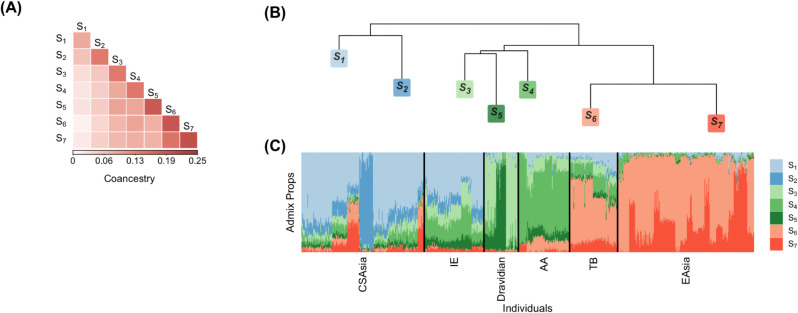
Visualization of population-level coancestry and admixture proportions in the merged data set of mainland Indians from IND, and Central/South Asians and East Asians from HGDP. (A) Heatmap of antecedent population coancestry estimates. (B) Dendrogram representation of the antecedent population coancestry estimates. (C) Stacked bar plot of admixture proportions.

Our analysis reveals that there are three major branches of antecedent populations for this data set (Fig 7). The branch of antecedent populations *S*_1_ and *S*_2_ is most prevalent in Central/South Asians of HGDP and Indo-European speakers, suggesting this branch was at least partially derived from a West Eurasian source. The branch of the antecedent populations *S*_3_, *S*_4_ and *S*_5_ is widespread in Dravidian speakers and Austro-Asiatic speakers, indicating it is relevant to South Indian ancestry and Austro-Asiatic speaker ancestry. The third branch of the antecedent populations *S*_6_ and *S*_7_ likely represents East Asian ancestry due to its high prevalence in the Tibeto-Burman speakers and East Asians of HGDP.

## Discussion

The super admixture framework is an extension of the highly used admixture model. It superposes coancestry among the admixed antecedent populations. It provides a forward-generating probability process that encompasses random evolutionary, genealogical, and statistical sampling processes. The antecedent populations are modeled to have an arbitrarily complex coancestry. This allows the generation of individual-specific allele frequencies (IAFs) that capture complex population structures and permit the estimation of individual-level coancestry that is at the resolution of general individual-level coancestry and kinship estimators for arbitrarily complex structures.

There are numerous parameters estimated from genome-wide genotype data that relate to structure, such as coancestry, inbreeding, and $F_{\text{ST}}$. When traits are included, one often estimates parameters in the context of genome-wide association studies [25,39], genome-wide heritability [40–42] and polygenic risk scores [43,44]. Within our framework, we have shown how to perform a bootstrap resampling method that randomly generates new genetic data that recapitulate population structure observed in real data. This bootstrap method may provide a way to formulate general methods for quantifying uncertainty in genome-wide genotype studies.

We proposed a hypothesis test for comparing the standard and super admixture models on real data. Additional work will be required to fully assess its validity, characterize its statistical power, and determine the conditions under which it yields reliable inference. When we applied it to the five data sets analyzed here, all of them were highly significant in rejecting the standard admixture model in favor of the super admixture model. The individual-level coancestry estimates from the super admixture model also agreed with the general coancestry estimate, whereas the standard admixture individual-level coancestry estimates did not.

As with any admixture method, the results of the super admixture framework should be interpreted with thoughtful consideration to ensure conclusions remain well supported. Similar to other admixture models, the super admixture model assumes each individual inherits ancestry from one or more of *K* antecedent populations. Real human population histories are usually complex and often involve processes such as recent gene flow, migrations, bottlenecks, and other events that do not fit neatly into discrete divergence and admixture phases. Consequently, we view the inferred antecedent populations as model-based components rather than direct representations of real human populations. Likewise, the dendrograms should be interpreted as a parsimonious summary of relationships among antecedent populations, rather than a literal reconstruction of population history. To obtain biologically interpretable source populations, it is essential to assess whether the assumptions underlying the admixture model are met, particularly whether a sufficiently large value of *K* has been used. In practice, we recommend exploring a wide range of *K* values and employing diagnostic tools to evaluate model fit [45,46].

Some sampling strategies can potentially bias the inference of population structure, a problem shared with other methods modeling structure [45]. The estimator that we utilized for individual coancestry estimation can accommodate biased sampling schemes by incorporating weights assigned to individuals in the study [4]. Alternatively, users may manually downsample individuals to achieve a more balanced representation across populations. These strategies can partially mitigate the impact of biased sampling on the inferred structure, but they do not eliminate the issue entirely. It therefore remains important to evaluate the overall robustness of the inferred structure in the presence of such sampling imbalances.

Understanding population structure in humans is one of the central problems in modern genetics. We demonstrated that the proposed super admixture framework is a powerful tool for learning admixed population coancestry, improving the analysis of genetic data from structured populations, bridging admixture with individual-level coancestry and kinship, and simulating new data reflecting a structured population. We anticipate that the super admixture framework will be useful in analyzing complex population structure in future applications.

## Supporting information

S1 Text A file containing supplementary information on the theory, methods, algorithms, numerical studies, and additional analyses of human studies. Appendices A–B: Mathematical definitions and lemmas supporting the algorithms; Appendices C–G: Supplementary methods; Appendices H–M: Supplementary simulations; Appendices N–U: Supplementary analyses of human studies.
(PDF)
