# Supplementary material for: Coancestry superposed on admixed populations yields measures of relatedness at individual-level resolution
Source: PLoS Comput Biol. 2025 Dec 31;21(12):e1013848. doi: 10.1371/journal.pcbi.1013848 (PMC12779161; doi:10.1371/journal.pcbi.1013848)
Supplement: S1 Text — Appendices A–B: Mathematical definitions and lemmas supporting the algorithms; Appendices C–G: Supplementary methods; Appendices H–M: Supplementary simulations; Appendices N–U: Supplementary analyses of human studies. (PDF) [file pcbi.1013848.s001.pdf]

Supplementary Information: Coancestry superposed on admixed populations yields measures of relatedness at individual-level resolution

Danfeng Chen[1], John D. Storey[1*]

1 Lewis-Sigler Institute for Integrative Genomics, Princeton University, Princeton, New Jersey, United States of America

* jstorey@princeton.edu

Contents

SUPPLEMENTARY THEORY **3**

A Mathematical notation and definitions **3**

B Lemmas supporting the algorithms **4**

SUPPLEMENTARY METHODS **7**

C Estimating individual-level coancestry **7**

D Estimating coancestry among antecedent populations **8**

E Estimating parameters in the double-admixture algorithm **10**

F Simulating antecedent population coancestry through NORmal To Anything (NORTA) **15**

G Constructing a dendrogram from population coancestry **17**

SUPPLEMENTARY SIMULATIONS **18**

H Generating $\boldsymbol{\Lambda}$ **18**

I Generating $\boldsymbol{Q}$ **18**

J Evaluating algorithms for estimating coancestry among antecedent populations **18**

K Evaluating algorithms for generating antecedent population allele frequencies **19**

L Evaluating the algorithm for generating genotypes from the super admixture model **20**

M Null p-value distribution of the hypothesis test of standard admixture versus super admixture **22**

SUPPLEMENTARY ANALYSES OF HUMAN STUDIES **23**

N Data processing **23**

O HGDP study analysis **25**

P TGP study analysis **27**

Q Confirming significant hypothesis tests of standard admixture versus super admixture in the human studies **28**

R Comparing the individual-level coancestry estimates **30**

S Selecting the number of antecedent populations **31**

T Analysis of HO over a range of antecedent population numbers **33**

U Analysis of IND over a range of antecedent population numbers **35**

References **38**

SUPPLEMENTARY THEORY

A Mathematical notation and definitions

Definition 1. Given a vector $\boldsymbol{x} \in \mathbb{R}^n$, the ℓ_p norm is defined as:

$$\|\boldsymbol{x}\|_p = \left(\sum_{i=1}^{n} |x_i|^p \right)^{1/p}.$$

Definition 2. Given a matrix $\boldsymbol{A} \in \mathbb{R}^{m \times n}$, the maximum singular value of $\boldsymbol{A}$ is denoted as $\sigma_{\max}(\boldsymbol{A})$. Given a matrix $\boldsymbol{A} \in \mathbb{R}^{n \times n}$, the maximum and minimum eigenvalues of $\boldsymbol{A}$ are denoted as $\lambda_{\max}(\boldsymbol{A})$ and $\lambda_{\min}(\boldsymbol{A})$.

Definition 3. Given a matrix $\boldsymbol{A} \in \mathbb{R}^{m \times n}$, the Frobenius norm of $\boldsymbol{A}$ is defined by:

$$\|\boldsymbol{A}\|_F = \sqrt{\operatorname{tr}(\boldsymbol{A}'\boldsymbol{A})} = \sqrt{\sum_{i=1}^{m} \sum_{j=1}^{n} a_{ij}^2}.$$

Definition 4. The induced matrix norm $\|\boldsymbol{A}\|_{a,b}$ is defined as:

$$\|\boldsymbol{A}\|_{a,b} = \sup\{\|\boldsymbol{A}\boldsymbol{x}\|_b : \|\boldsymbol{x}\|_a = 1\}.$$

When $a = b = 2$, the induced matrix norm is the spectral norm:

$$\|\boldsymbol{A}\|_2 \equiv \|\boldsymbol{A}\|_{2,2} = \sqrt{\lambda_{\max}(\boldsymbol{A}'\boldsymbol{A})} = \sigma_{\max}(\boldsymbol{A}).$$

When $a = b = 1$, the induced matrix norm is the maximum absolute column sum of the matrix:

$$\|\boldsymbol{A}\|_1 \equiv \|\boldsymbol{A}\|_{1,1} = \max_j \sum_{i=1}^{m} |a_{ij}|.$$

Definition 5. The proximal operator $\operatorname{prox}_f(\boldsymbol{x})$ is defined as

$$\operatorname{prox}_f(\boldsymbol{x}) = \arg\min_{\boldsymbol{u} \in \mathbb{R}^n} f(\boldsymbol{u}) + \frac{1}{2}\|\boldsymbol{u} - \boldsymbol{x}\|_2^2.$$

Let $f(\boldsymbol{x})$ be an indicator function defined as

$$1_C(\boldsymbol{x}) = \begin{cases} 0 & \boldsymbol{x} \in C \\ \infty & \boldsymbol{x} \notin C \end{cases},$$

where C is a nonempty subset $\mathbb{R}^n$. Let $\mathcal{P}_C$ denote the "projection onto C operator". Then

$$\text{prox}_f(\boldsymbol{x}) = \underset{\boldsymbol{u} \in C}{\arg\min} \|\boldsymbol{u} - \boldsymbol{x}\|_2^2 = \mathcal{P}_C(\boldsymbol{x}).$$

The n-dimensional unit simplex is defined as

$$\Delta = \left\{ \boldsymbol{x} \in \mathbb{R}^n : \sum_{i=1}^{n} x_i = 1, 0 \leq x_i \leq 1 \right\}.$$

We define the projection onto the unit simplex operator $\mathcal{P}_\Delta$ as

$$\mathcal{P}_\Delta(\boldsymbol{x}) = \underset{\boldsymbol{u} \in \Delta}{\arg\min} \|\boldsymbol{u} - \boldsymbol{x}\|_2^2.$$

Definition 6. A subset S of $\mathbb{R}^n$ is a real semi-algebraic set if there exists a finite number of real polynomial functions g_{ij}, h_{ij}: $\mathbb{R}^n \to \mathbb{R}$ such that

$$S = \cup_{j=1}^{p} \cap_{i=1}^{q} \{\boldsymbol{u} : \mathbb{R}^n : g_{ij}(\boldsymbol{u}) = 0 \text{ and } h_{ij}(\boldsymbol{u}) < 0\}.$$

Definition 7. A function $f : \mathbb{R}^n \to (-\infty, \infty]$ is called semi-algebraic if its graph

$$\{(\boldsymbol{u}, t) \in \mathbb{R}^{n+1} : f(\boldsymbol{u}) = t\}$$

is a semi-algebraic subset of $\mathbb{R}^{n+1}$.

B Lemmas supporting the algorithms

Lemma 1. The definition of the induced matrix norm $\|\boldsymbol{A}\|_{a,b}$ implies that for any $\boldsymbol{x} \in \mathbb{R}^n$, the following sub-additivity property holds:

$$\|\boldsymbol{A}\boldsymbol{x}\|_b \leq \|\boldsymbol{A}\|_{a,b} \|\boldsymbol{x}\|_a.$$

Lemma 2. Denote the collection of $\mathbb{R}^{n \times n}$ non-negative definite symmetric matrices as $\mathbb{S}_+^n$.

Given the matrices $\boldsymbol{A}, \boldsymbol{B} \in \mathbb{S}_+^n$,

$$\lambda_{\min}(\boldsymbol{A})\text{tr}(\boldsymbol{B}) \leq \text{tr}(\boldsymbol{AB}) \leq \lambda_{\max}(\boldsymbol{A})\text{tr}(\boldsymbol{B}).$$

Proof. Since $\boldsymbol{A} \in \mathbb{S}_+^n$, there is an orthogonal matrix $\boldsymbol{U}$ such that $\boldsymbol{U}'\boldsymbol{AU} = \boldsymbol{\Lambda}$. Then

$$\text{tr}(\boldsymbol{AB}) = \text{tr}(\boldsymbol{ABUU}') = \text{tr}(\boldsymbol{U}'\boldsymbol{AUU}'\boldsymbol{BU}) = \text{tr}(\boldsymbol{\Lambda}\tilde{\boldsymbol{B}}),$$

where $\tilde{\boldsymbol{B}} = \boldsymbol{U}'\boldsymbol{BU}$. Observe that the diagonal element of $\boldsymbol{\Lambda}\tilde{\boldsymbol{B}}$ satisfies

$$\begin{aligned}
[\boldsymbol{\Lambda}\tilde{\boldsymbol{B}}]_{ii} &= \sum_{j=1}^{n} \lambda_{ij}\tilde{b}_{ji} = \lambda_{ii}\tilde{b}_{ii} && (\boldsymbol{\Lambda} \text{ diagonal}) \\
&= \lambda_{ii}\boldsymbol{u}_i'\boldsymbol{B}\boldsymbol{u}_i && (\boldsymbol{u}_i \text{ denotes } i\text{th column of } \boldsymbol{U}) \\
&\leq \lambda_{\max}(\boldsymbol{A})\boldsymbol{u}_i'\boldsymbol{B}\boldsymbol{u}_i.
\end{aligned}$$

Following this, it follows that

$$\begin{aligned}
\text{tr}(\boldsymbol{AB}) &= \sum_{i=1}^{n}[\boldsymbol{\Lambda}\tilde{\boldsymbol{B}}]_{ii} \leq \sum_{i=1}^{n}\lambda_{\max}(\boldsymbol{A})\boldsymbol{u}_i'\boldsymbol{B}\boldsymbol{u}_i \\
&= \lambda_{\max}(\boldsymbol{A})\text{tr}(\boldsymbol{B}\sum_{i=1}^{n}\boldsymbol{u}_i\boldsymbol{u}_i') = \lambda_{\max}(\boldsymbol{A})\text{tr}(\boldsymbol{BUU}') = \lambda_{\max}(\boldsymbol{A})\text{tr}(\boldsymbol{B}).
\end{aligned}$$

Similarly, we can show that $\lambda_{\min}(\boldsymbol{A})\text{tr}(\boldsymbol{B}) \leq \text{tr}(\boldsymbol{AB})$. □

Lemma 3. Given matrices $\boldsymbol{A}, \boldsymbol{B} \in \mathbb{R}^{m\times n}$,

$$\sigma_{\min}(\boldsymbol{A})\|\boldsymbol{B}\|_F \leq \|\boldsymbol{AB}\|_F \leq \sigma_{\max}(\boldsymbol{A})\|\boldsymbol{B}\|_F = \|\boldsymbol{A}\|_2\|\boldsymbol{B}\|_F.$$

Proof. We note that

$$\|\boldsymbol{AB}\|_F^2 = \text{tr}(\boldsymbol{ABB}'\boldsymbol{A}') = \text{tr}(\boldsymbol{A}'\boldsymbol{AB}'\boldsymbol{B}).$$

Since $\boldsymbol{A}'\boldsymbol{A}, \boldsymbol{B}'\boldsymbol{B} \in \mathbb{S}_+^n$, Lemma 2 implies

$$\lambda_{\min}(\boldsymbol{A}'\boldsymbol{A})\text{tr}(\boldsymbol{BB}) \leq \|\boldsymbol{AB}\|_F^2 \leq \lambda_{\max}(\boldsymbol{A}'\boldsymbol{A})\text{tr}(\boldsymbol{BB}).$$

Observing that $\mathrm{tr}(\boldsymbol{B}'\boldsymbol{B}) = \|\boldsymbol{B}\|_F^2$, it follows that

$$\sigma_{\min}(\boldsymbol{A})\|\boldsymbol{B}\|_F \leq \|\boldsymbol{AB}\|_F \leq \sigma_{\max}(\boldsymbol{A})\|\boldsymbol{B}\|_F = \|\boldsymbol{A}\|_2\|\boldsymbol{B}\|_F.$$

□

Lemma 4. Given a matrix $\boldsymbol{A} \in \mathbb{R}^{m\times n}$,

$$\frac{1}{m}\|\boldsymbol{A}\|_1 \leq \|\boldsymbol{A}\|_2 \leq \sqrt{n}\|\boldsymbol{A}\|_1.$$

Proof. Using the Cauchy-Schwartz inequality, it follows that for all $\boldsymbol{x} \in \mathbb{R}^n$,

$$\|\boldsymbol{x}\|_1 = \sum_{i=1}^n |x_i| = \sum_{i=1}^n |x_i| \cdot 1 \leq \left(\sum_{i=1}^n |x_i|^2\right)^{1/2} \left(\sum_{i=1}^n 1^2\right)^{1/2} = \sqrt{n}\|\boldsymbol{x}\|_2.$$

Also, by expanding the product $(\sum_i |x_i|)^2 = \sum_i |x_i|^2 + \sum_{i\neq j} |x_i||x_j|$ where all cross terms $|x_i||x_j| \geq 0$,

$$\|\boldsymbol{x}\|_2^2 = \sum_{i=1}^n |x_i|^2 \leq \left(\sum_{i=1}^n |x_i|\right)^2 = \|\boldsymbol{x}\|_1^2.$$

For $\boldsymbol{x} \in \mathbb{R}^n$,

$$\|\boldsymbol{Ax}\|_2 \leq \|\boldsymbol{Ax}\|_1 \leq \|\boldsymbol{A}\|_1\|\boldsymbol{x}\|_1 \leq \|\boldsymbol{A}\|_1\sqrt{n}\|\boldsymbol{x}\|_2,$$

where $\|\boldsymbol{Ax}\|_1 \leq \|\boldsymbol{A}\|_1\|\boldsymbol{x}\|_1$ is from the sub-additivity of the induced norm defined in Lemma 1. By the definition of matrix ℓ_2 norm,

$$\|\boldsymbol{A}\|_2 = \sup\{\|\boldsymbol{Ax}\|_2 : \|\boldsymbol{x}\|_2 = 1\} \leq \sup\{\sqrt{n}\|\boldsymbol{A}\|_1\|\boldsymbol{x}\|_2 : \|\boldsymbol{x}\|_2 = 1\} = \sqrt{n}\|\boldsymbol{A}\|_1.$$

□

Lemma 5. Let $f : \mathbb{R}^n \to (-\infty, \infty]$ be a proper and closed function. If f is semi-algebraic then it satisfies the Kurdyka-Lojasiewicz (KL) property at any point of $\mathrm{dom}(f)$ [1].

SUPPLEMENTARY METHODS

C Estimating individual-level coancestry

Here we briefly summarize the OS estimate of coancestry from [2], which we computed using the R package `popkin` [3]. The estimate begins with an allele matching measurement between each pair of individuals:

$$A_{jk} = \frac{1}{m} \sum_{i=1}^{m} (x_{ij} - 1)(x_{ik} - 1) - 1.$$

The expectation of A_{jk} is

$$\mathbb{E}[A_{jk}] = \begin{cases} \frac{(\theta_{jj}-1)\nu}{2} & j = k \\ (\theta_{jk} - 1)\nu & j \neq k \end{cases},$$

where $\nu = \frac{4}{m} \sum_{i=1}^{m} a_i(1 - a_i)$. Under the assumptions outlined in ref. [2], it is also the case that $A_{jk} \xrightarrow[m\to\infty]{a.s.} \mathbb{E}[A_{jk}]$.

Let $\underline{A} = \min_{j \neq k} \mathbb{E}[A_{jk}]$ and $\underline{\theta} = \min_{j \neq k} \theta_{jk}$. Since $(\theta_{jk} - 1)\nu = \mathbb{E}[A_{jk}]$ for all j and k, it follows that $(\underline{\theta} - 1)\nu = \underline{A}$ and $\nu = -\underline{A}/(1 - \underline{\theta})$. Therefore,

$$\theta_{jk} = \begin{cases} 1 - \frac{2(1-\underline{\theta})\mathbb{E}[A_{jj}]}{\underline{A}} & j = k \\ 1 - \frac{(1-\underline{\theta})\mathbb{E}[A_{jk}]}{\underline{A}} & j \neq k \end{cases}.$$

Given that $A_{jk} \xrightarrow[m\to\infty]{as} \mathbb{E}[A_{jk}]$, it follows that A_{jk} serves as a method of moments estimator of $\mathbb{E}[A_{jk}]$. The OS estimate utilizes $\underline{\theta} = 0$, which sets the reference population T to the most recent common ancestral (MRCA) population [2]. When $\underline{\theta} = 0$, then $\nu = -\underline{A}$. Given an estimate $\hat{\underline{A}}$ of $\underline{A}$, the OS estimator of coancestry is

$$\hat{\theta}^{\text{OS}}_{jk} = \begin{cases} 1 - \frac{2A_{jj}}{\hat{\underline{A}}} & j = k \\ 1 - \frac{A_{jk}}{\hat{\underline{A}}} & j \neq k \end{cases}.$$

The estimator $\hat{\underline{A}} = \min_{j \neq k} A_{jk}$ can be a consistent estimator of $\underline{A}$ as $m \to \infty$ under certain assumptions. However, for finite m, the variance could be large or the estimator could be susceptible to outliers. To address this, an estimator considered in ref. [2] relies on having available a partition of the n individuals into K sub-populations (denoted by S_u for

$u \in \{1, \ldots, K\}$):

$$\hat{\underline{A}} = \min_{u \neq v} \frac{1}{|S_u||S_v|} \sum_{j \in S_u} \sum_{k \in S_v} A_{jk}.$$

If the coancestry coefficients between all pairs of individuals from the two most unrelated subpopulations is zero, then $\hat{\underline{A}}$ could also be consistent. When no partition is available, then each individual is its own subpopulation, and these two estimators are equal. For all data sets, except AMR, we had subpopulation labels available and used the latter estimate of $\underline{A}$.

Note that since $\nu = -\underline{A}/(1 - \underline{\theta})$, one can extend the OS estimator of coancestry for general values of $\underline{\theta}$ as follows:

$$\hat{\theta}^{\mathrm{OS}}_{jk} = \begin{cases} 1 - \frac{2(1-\underline{\theta})A_{jj}}{\hat{\underline{A}}} & j = k \\ 1 - \frac{(1-\underline{\theta})A_{jk}}{\hat{\underline{A}}} & j \neq k \end{cases}.$$

The methods proposed here are compatible with this extended estimator as well as other valid estimators of coancestry.

D Estimating coancestry among antecedent populations

Proximal Forward-Backward (PFB) algorithm. Let $f : \mathbb{R}^n \to (-\infty, +\infty]$ be a be proper and closed function, let $h : \mathbb{R}^n \to (-\infty, +\infty)$ be convex and differentiable with a L-Lipschitz continuous gradient ∇h, i.e.,

$$\|\nabla h(\boldsymbol{x}_2) - \nabla h(\boldsymbol{x}_1)\|_2 \leq L\|\boldsymbol{x}_2 - \boldsymbol{x}_1\|_2 \quad \forall(\boldsymbol{x}_1, \boldsymbol{x}_2),$$

where $L \in (0, \infty)$. Suppose that $f(\boldsymbol{x}) + h(\boldsymbol{x}) \to \infty$ as $\|\boldsymbol{x}\|_2 \to \infty$. The problem is to identify:

$$\arg\min_{\boldsymbol{x} \in \mathbb{R}^n} f(\boldsymbol{x}) + h(\boldsymbol{x}).$$

It has been shown that this problem can be solved by the PFB algorithm [4]. Every sequence $(\boldsymbol{x}_t)_{t \in \mathbb{N}}$ generated by the following constant-step forward-backward algorithm converges to a solution to the problem.

Alg A: The constant-step forward-backward algorithm

Initialize $\boldsymbol{x}_0$

for $t = 1, 2, \ldots$ **do**

  $\boldsymbol{x}^* \leftarrow \boldsymbol{x}_{t-1} - \frac{1}{L}\nabla h(\boldsymbol{x}_{t-1})$

  $\boldsymbol{x}_t \leftarrow \text{prox}_{L^{-1}f}(\boldsymbol{x}^*)$

return $\boldsymbol{x}_t$

prox$(\cdot)$ denotes the proximal operator (Appendix A).

Solving Problem 1 by PFB. Problem 1 is equivalent to the problem of finding the minimizer of $f(\boldsymbol{\Lambda}) + h(\boldsymbol{\Lambda})$ where

$$f(\boldsymbol{\Lambda}) = \begin{cases} 0 & \boldsymbol{\Lambda} \text{ is symmetric and } 0 \leq \lambda_{uv} \leq 1, u, v = 1, \ldots, K \\ \infty & \text{otherwise} \end{cases}$$
$$h(\boldsymbol{\Lambda}) = \|\boldsymbol{\Theta} - \boldsymbol{Q}'\boldsymbol{\Lambda}\boldsymbol{Q}\|_F^2.$$

The function f is proper and closed because $\text{dom}(f)$ is nonempty and closed. The function h is differentiable with a continuous gradient $\nabla h = -2\boldsymbol{Q}(\boldsymbol{\Theta} - \boldsymbol{Q}'\boldsymbol{\Lambda}\boldsymbol{Q})\boldsymbol{Q}'$. ∇h is Lipschitz continuous with $L = \sigma_{\max}^4(\boldsymbol{Q})$.

Proof. We note that

$$\begin{aligned} \|\nabla h(\boldsymbol{\Lambda}_2) - \nabla h(\boldsymbol{\Lambda}_1)\|_F &= \| - 2\boldsymbol{Q}(\boldsymbol{\Theta} - \boldsymbol{Q}'\boldsymbol{\Lambda}_2\boldsymbol{Q})\boldsymbol{Q}' + -2\boldsymbol{Q}(\boldsymbol{\Theta} - \boldsymbol{Q}'\boldsymbol{\Lambda}_1\boldsymbol{Q})\boldsymbol{Q}'\|_F \\ &= 2\|\boldsymbol{Q}\boldsymbol{Q}'(\boldsymbol{\Lambda}_2 - \boldsymbol{\Lambda}_1)\boldsymbol{Q}\boldsymbol{Q}'\|_F \leq 2\sigma_{\max}^2(\boldsymbol{Q}\boldsymbol{Q}')\|\boldsymbol{\Lambda}_2 - \boldsymbol{\Lambda}_1\|_F \quad (\textit{Lemma } 3) \\ &= 2\sigma_{\max}^4(\boldsymbol{Q})\|\boldsymbol{\Lambda}_2 - \boldsymbol{\Lambda}_1\|_F \end{aligned}$$

□

Therefore, we can employ the PFB algorithm to solve Problem 1. The proximal operator $\text{prox}_{L^{-1}f}(\boldsymbol{\Lambda})$ can be calculated as $\text{prox}_{L^{-1}f}(\boldsymbol{\Lambda}) = \mathcal{P}_{\text{dom}(f)}(\boldsymbol{\Lambda})$. This implies

$$\{\text{prox}_{L^{-1}f}(\boldsymbol{\Lambda})\}_{uv} = \max(0, \min(\lambda_{uv}, 1)).$$

This leads to Alg 1, where we have now proved that every sequence $(\boldsymbol{\Lambda}_t)_{t\in\mathbb{N}}$ converges to a solution.

Solving Problem A by PFB. We can formulate the estimation of coancestry among antecedent populations under the standard admixture model as follows.

Problem A.

$$\begin{aligned} &\min_{\boldsymbol{\Lambda} \in \mathbb{R}^{K \times K}} \|\boldsymbol{\Theta} - \boldsymbol{Q}'\boldsymbol{\Lambda}\boldsymbol{Q}\|_F^2 \\ \text{subject to: } &0 \leq \lambda_{uu} \leq 1 \\ &\lambda_{uv} = 0 \quad \forall u \neq v \\ &u, v = 1, 2, \ldots, K \end{aligned}$$

It is straightforward to see that Problem A is identical to identifying the minimizer of $f(\boldsymbol{\Lambda}) + h(\boldsymbol{\Lambda})$ where

$$f(\boldsymbol{\Lambda}) = \begin{cases} 0 & 0 \leq \lambda_{uu} \leq 1; \lambda_{uv} = 0, \forall u \neq v; u, v = 1, \ldots, K \\ \infty & \text{otherwise} \end{cases},$$

$$h(\boldsymbol{\Lambda}) = \|\boldsymbol{\Theta} - \boldsymbol{Q}'\boldsymbol{\Lambda}\boldsymbol{Q}\|_F^2.$$

The function f is proper and closed and h is differentiable with a continuous gradient $\nabla h = -2\boldsymbol{Q}(\boldsymbol{\Theta} - \boldsymbol{Q}'\boldsymbol{\Lambda}\boldsymbol{Q})\boldsymbol{Q}'$. The gradient ∇h is Lipschitz continuous with $L = \sigma_{\max}^4(\boldsymbol{Q})$. By Appendix A, $\text{prox}_{L^{-1}f}(\boldsymbol{\Lambda}) = \mathcal{P}_{\text{dom}(f)}(\boldsymbol{\Lambda})$. This implies:

$$\{\text{prox}_{L^{-1}f}(\boldsymbol{\Lambda})\}_{uv} = \begin{cases} \max(0, \min(\lambda_{uu}, 1)) & u = v \\ 0 & u \neq v \end{cases}.$$

This leads to Alg B, where every sequence $(\boldsymbol{\Lambda}_t)_{t \in \mathbb{N}}$ converges to a solution.

E Estimating parameters in the double-admixture algorithm

Proximal Alternating Linearized Minimization (PALM) agloriithm. Let $f : \mathbb{R}^n \to (-\infty, +\infty]$ and $g : \mathbb{R}^m \to (-\infty, +\infty)$ be closed functions. Let $h : \mathbb{R}^n \times \mathbb{R}^m \to \mathbb{R}$ be a continuously differentiable function. The problem is to find a solution to:

$$\arg\min_{\boldsymbol{x} \in \mathbb{R}^n, \boldsymbol{y} \in \mathbb{R}^m} \Psi(\boldsymbol{x}, \boldsymbol{y}) = f(\boldsymbol{x}) + g(\boldsymbol{y}) + h(\boldsymbol{x}, \boldsymbol{y})$$

Alg B: Estimating $\boldsymbol{\Lambda}$ for the standard admixture model given $\boldsymbol{\Theta}$ and $\boldsymbol{Q}$

input: Coancestry matrix $\boldsymbol{\Theta}$ and admixture proportions matrix $\boldsymbol{Q}$

let $L = \sigma^4_{\max}(\boldsymbol{Q})$

let $\boldsymbol{\Lambda}_0 \leftarrow (\boldsymbol{Q}\boldsymbol{Q}')^{-1}\boldsymbol{\Theta}(\boldsymbol{Q}\boldsymbol{Q}')^{-1}$

for $t = 1, 2, \ldots$ **do**

  $\boldsymbol{G} \leftarrow 2\boldsymbol{Q}(\boldsymbol{Q}'\boldsymbol{\Lambda}_{t-1}\boldsymbol{Q} - \boldsymbol{\Theta})\boldsymbol{Q}'$

  $\boldsymbol{\Lambda}^* \leftarrow \boldsymbol{\Lambda}_{t-1} - \frac{1}{L}\boldsymbol{G}$

  $\boldsymbol{\Lambda}_t = \{\lambda_{uv,t}\}$ where

$$\lambda_{uv,t} = \begin{cases} \max(0, \min(1, \lambda^*_{uu})), & \text{if } u = v \\ 0, & \text{if } u \neq v \end{cases}$$

return $\boldsymbol{\Lambda}_t$

$\sigma_{\max}(\cdot)$ denotes the maximum singular value (Appendix A).

over all $(\boldsymbol{x}, \boldsymbol{y}) \in \mathbb{R}^n \times \mathbb{R}^m$. It has been shown that this problem can be solved by the Proximal Alternating Linearized Minimization (PALM) algorithm. Assume that:

(i) $\inf_{\mathbb{R}^n \times \mathbb{R}^m} \Psi > -\infty$, $\inf_{\mathbb{R}^n} f > -\infty$ and $\inf_{\mathbb{R}^m} g > -\infty$.

(ii) Ψ is a Kurdyka-Lojasiewicz function (see Appendix A).

(iii) h is twice continuously differentiable.

(iv) There exists convex and compact sets $\mathcal{C}_{\boldsymbol{x}}$ and $\mathcal{C}_{\boldsymbol{y}}$ such that $\boldsymbol{x}_t \in \mathcal{C}_{\boldsymbol{x}}$ and $\boldsymbol{y}_t \in \mathcal{C}_{\boldsymbol{y}}$ for all $t \in \mathbb{N}$.

(v) For any fixed $\boldsymbol{y}$ the partial gradient $\nabla_{\boldsymbol{x}} h(\boldsymbol{x}, \boldsymbol{y})$ is Lipschitz continuous with moduli $L_1(\boldsymbol{y})$ over the domain $\mathcal{C}_{\boldsymbol{x}}$, that is

$$\|\nabla_{\boldsymbol{x}} h(\boldsymbol{x}_1, \boldsymbol{y}) - \nabla_{\boldsymbol{x}} h(\boldsymbol{x}_2, \boldsymbol{y})\|_2 \leq L_1(\boldsymbol{y})\|\boldsymbol{x}_1 - \boldsymbol{x}_2\|_2, \quad \forall \boldsymbol{x}_1, \boldsymbol{x}_2 \in \mathcal{C}_{\boldsymbol{x}}.$$

Likewise, for any fixed $\boldsymbol{x}$ the partial gradient $\nabla_{\boldsymbol{y}} h(\boldsymbol{x}, \boldsymbol{y})$ is Lipschitz continuous with moduli $L_2(\boldsymbol{x})$ over the domain $\mathcal{C}_{\boldsymbol{y}}$.

(vi) For $i = 1, 2$ there exists $\lambda_i^-, \lambda_i^+ > 0$ such that:

$$\inf\{L_1(\boldsymbol{y}_t) : t \in \mathbb{N}\} \geq \lambda_1^-$$
$$\inf\{L_2(\boldsymbol{x}_t) : t \in \mathbb{N}\} \geq \lambda_2^-$$
$$\sup\{L_1(\boldsymbol{y}_t) : t \in \mathbb{N}\} \leq \lambda_1^+$$
$$\sup\{L_2(\boldsymbol{x}_t) : t \in \mathbb{N}\} \leq \lambda_2^+$$

We note that these assumptions are not exactly the same as the assumptions specified in ref. [1]. We modified the original assumptions to align the PALM algorithm to our setting. Following the proof provided in ref. [1], one can show that if these assumptions are met, the sequence $(\boldsymbol{x}_t, \boldsymbol{y}_t)_{t\in\mathbb{N}}$ generated by Alg C converges to a critical point of Ψ.

Alg C: The general PALM algorithm

Initialization: start with any $\boldsymbol{x}_0 \in \mathcal{C}_{\boldsymbol{x}}$ and $\boldsymbol{y}_0 \in \mathcal{C}_{\boldsymbol{y}}$
for $t = 1, 2, \ldots$ **do**
  take $\tau_1 > 1$ and set $c = \tau_1 L_1(\boldsymbol{y}_{t-1})$ and compute
    $\boldsymbol{x}_t = \text{prox}_c^f\left(\boldsymbol{x}_{t-1} - \frac{1}{c}\nabla_{\boldsymbol{x}} h(\boldsymbol{x}_{t-1}, \boldsymbol{y}_{t-1})\right)$
  take $\tau_1 > 1$ and set $d = \tau_2 L_2(\boldsymbol{x}_t)$ and compute
    $\boldsymbol{y}_t = \text{prox}_d^g\left(\boldsymbol{y}_{t-1} - \frac{1}{d}\nabla_{\boldsymbol{y}} h(\boldsymbol{x}_t, \boldsymbol{y}_{t-1})\right)$

prox$(\cdot)$ denotes the proximal operator (Appendix A).

Solving Problem 2 by PALM. Problem 2 is identical to identifying the minimizer of $f(\boldsymbol{W}) + g(\boldsymbol{\Gamma}) + h(\boldsymbol{W}, \boldsymbol{\Gamma})$ where

$$f(\boldsymbol{W}) = \begin{cases} 0 & \boldsymbol{W} \in \mathbb{R}^{S\times K} : 0 \leq w_{su} \leq 1, \sum_{s=1}^S w_{su} = 1 \\ \infty & \text{otherwise} \end{cases},$$

$$g(\boldsymbol{\Gamma}) = \begin{cases} 0 & \boldsymbol{\Gamma} \in \mathbb{R}^{S\times S} : \epsilon \leq \gamma_{ss} \leq 1 - \epsilon; \gamma_{ss'} = 0\ \forall s \neq s' \\ \infty & \text{otherwise} \end{cases},$$

and h is defined as $h(\boldsymbol{W}, \boldsymbol{\Gamma}) = \|\boldsymbol{\Lambda} - \boldsymbol{W}'\boldsymbol{\Gamma}\boldsymbol{W}\|_F^2$. Define $\mathcal{C}_{\boldsymbol{W}} = \{\boldsymbol{W} \in \mathbb{R}^{S\times K} : w_{su} \geq 0, \sum_{s=1}^S w_{su} = 1\}$. Define $\mathcal{C}_{\boldsymbol{\Gamma}} = \{\boldsymbol{\Gamma} \in \mathbb{R}^{S\times S} : \epsilon \leq \gamma_{ss} \leq 1 - \epsilon; \gamma_{ss'} = 0\ \forall s \neq s'\}$. We note that

both functions $\boldsymbol{W} \to \nabla_{\boldsymbol{W}} h(\boldsymbol{W}, \boldsymbol{\Gamma})$ and $\boldsymbol{\Gamma} \to \nabla_{\boldsymbol{\Gamma}} h(\boldsymbol{W}, \boldsymbol{\Gamma})$ are continuous. Indeed,

$$\nabla_{\boldsymbol{W}} h(\boldsymbol{W}, \boldsymbol{\Gamma}) = -4\boldsymbol{\Gamma}\boldsymbol{W}(\boldsymbol{\Lambda} - \boldsymbol{W}'\boldsymbol{\Gamma}\boldsymbol{W}), \tag{1}$$

$$\nabla_{\boldsymbol{\Gamma}} h(\boldsymbol{W}, \boldsymbol{\Gamma}) = -2\boldsymbol{W}(\boldsymbol{\Lambda} - \boldsymbol{W}'\boldsymbol{\Gamma}\boldsymbol{W})\boldsymbol{W}'. \tag{2}$$

For all $\boldsymbol{W}_1, \boldsymbol{W}_2 \in \mathcal{C}_{\boldsymbol{W}}$,

$$\|\nabla_{\boldsymbol{W}} h(\boldsymbol{W}_1, \boldsymbol{\Gamma}) - \nabla_{\boldsymbol{W}} h(\boldsymbol{W}_2, \boldsymbol{\Gamma})\|_F \leq 4(\|\boldsymbol{\Lambda}\|_2 \|\boldsymbol{\Gamma}\|_2 + 3K\|\boldsymbol{\Gamma}\|_2^2)\|\boldsymbol{W}_1 - \boldsymbol{W}_2\|_F. \tag{3}$$

For all $\boldsymbol{\Gamma}_1, \boldsymbol{\Gamma}_2 \in \mathcal{C}_{\boldsymbol{\Gamma}}$,

$$\|\nabla_{\boldsymbol{\Gamma}} h(\boldsymbol{W}, \boldsymbol{\Gamma}_1) - \nabla_{\boldsymbol{\Gamma}} h(\boldsymbol{W}, \boldsymbol{\Gamma}_2)\|_F \leq 2\|\boldsymbol{W}\|_2^4 \|\boldsymbol{\Gamma}_1 - \boldsymbol{\Gamma}_2\|_F. \tag{4}$$

Eqs (3) and (4) are proved in the following paragraphs. By Appendix A, $\mathrm{prox}_c^f(\boldsymbol{W}) = \mathcal{P}_{\mathrm{dom}(f)}(\boldsymbol{W})$ and $\mathrm{prox}_c^f(\boldsymbol{\Gamma}) = \mathcal{P}_{\mathrm{dom}(g)}(\boldsymbol{\Gamma})$. This implies

$$\{\mathrm{prox}_d^g(\boldsymbol{\Gamma})\}_{ss'} = \begin{cases} \min(\epsilon, \max(\gamma_{ss}, 1-\epsilon)) & s = s' \\ 0 & \text{otherwise} \end{cases} \tag{5}$$

and

$$\mathrm{prox}_c^f(\boldsymbol{W}) = \begin{pmatrix} \mathcal{P}_\Delta(\boldsymbol{w}_1) & \dots & \mathcal{P}_\Delta(\boldsymbol{w}_K) \end{pmatrix}, \tag{6}$$

where $\boldsymbol{w}_1, \dots, \boldsymbol{w}_K$ are columns of $\boldsymbol{W}$. Applying Eqs (1) to (6) to Alg C, we arrive at Alg 3 for solving Problem 2.

Proving the convergence of Alg 3. To prove the convergence, we need to show all assumptions of PALM hold. It is obvious that the assumptions (i), (iii) and (iv) hold.

Proof of assumption (ii): Ψ is a KL function. By Lemma 5, we note that the objective function H is a real polynomial function, hence semi-algebraic. For the indicator function f, we observe that the domain of f is defined by $\mathrm{dom}(f) = \cap_{u=1}^K \{\boldsymbol{W} \in \mathbb{R}^{S\times K} : \boldsymbol{w}_u'\boldsymbol{1} = 1 \text{ and } \boldsymbol{w}_u \geq \boldsymbol{0}\}$. Hence, $\mathrm{dom}(f)$ is a semi-algebraic set, so f is a semi-algebraic function. For the indicator function g, we observe that the domain of g is defined by $\mathrm{dom}(g) = \cap_{s=1}^S \{\boldsymbol{\Gamma} \in \mathbb{R}^{S\times S} : \epsilon \leq \boldsymbol{e}_s'\boldsymbol{\Gamma}\boldsymbol{e}_s \leq 1-\epsilon\}$, where $\boldsymbol{e}_1 = (1, 0, \dots, 0)$, …, $\boldsymbol{e}_S = (0, 0, \dots, 1)$. Hence, $\mathrm{dom}(g)$ is a semi-algebraic set, so g is a semi-algebraic function. Thus, $\Psi = f + g + H$ is a semi-algebraic function, and Ψ satisfies the KL property of any point of its domain.

Proof of assumption (v): To prove the assumption (v), we are to show Eqs (3) and (4). We note that for all $\boldsymbol{W} \in \mathcal{C}_{\boldsymbol{W}}$, by the definition of the induced matrix norm and Lemma 4, we have $\|\boldsymbol{W}\|_2 \leq \sqrt{K}\|\boldsymbol{W}\|_1 = \sqrt{K}$. For all $\boldsymbol{W}_1, \boldsymbol{W}_2 \in \mathcal{C}_{\boldsymbol{W}}$:

$$
\begin{aligned}
&\|\nabla_{\boldsymbol{W}} H(\boldsymbol{W}_1, \boldsymbol{\Gamma}) - \nabla_{\boldsymbol{W}} H(\boldsymbol{W}_2, \boldsymbol{\Gamma})\|_F \\
=&\| - 4\boldsymbol{\Gamma}\boldsymbol{W}_1(\boldsymbol{\Lambda} - \boldsymbol{W}_1'\boldsymbol{\Gamma}\boldsymbol{W}_1) + 4\boldsymbol{\Gamma}\boldsymbol{W}_2(\boldsymbol{\Lambda} - \boldsymbol{W}_2'\boldsymbol{\Gamma}\boldsymbol{W}_2)\|_F \\
\leq& 4\|\boldsymbol{\Gamma}(\boldsymbol{W}_1 - \boldsymbol{W}_2)\boldsymbol{\Lambda}\|_F + 4\|\boldsymbol{\Gamma}\boldsymbol{W}_1\boldsymbol{W}_1'\boldsymbol{\Gamma}\boldsymbol{W}_1 - \boldsymbol{\Gamma}\boldsymbol{W}_2\boldsymbol{W}_2'\boldsymbol{\Gamma}\boldsymbol{W}_2\|_F \\
\leq& 4\|\boldsymbol{\Gamma}\|_2\|\boldsymbol{\Lambda}\|_2\|\boldsymbol{W}_1 - \boldsymbol{W}_2\|_F + 4\underbrace{\|\boldsymbol{\Gamma}\boldsymbol{W}_1\boldsymbol{W}_1'\boldsymbol{\Gamma}\boldsymbol{W}_1 - \boldsymbol{\Gamma}\boldsymbol{W}_2\boldsymbol{W}_2'\boldsymbol{\Gamma}\boldsymbol{W}_2\|_F}_{*} \quad \text{(by Lemma 3)}
\end{aligned}
$$

$$
\begin{aligned}
(*) =& \|\boldsymbol{\Gamma}\boldsymbol{W}_1\boldsymbol{W}_1'\boldsymbol{\Gamma}\boldsymbol{W}_1 - \boldsymbol{\Gamma}\boldsymbol{W}_2\boldsymbol{W}_2'\boldsymbol{\Gamma}\boldsymbol{W}_2\|_F \\
=& \|\boldsymbol{\Gamma}\boldsymbol{W}_1\boldsymbol{W}_1'\boldsymbol{\Gamma}\boldsymbol{W}_1 - \boldsymbol{\Gamma}\boldsymbol{W}_2\boldsymbol{W}_1'\boldsymbol{\Gamma}\boldsymbol{W}_1 \\
&+ \boldsymbol{\Gamma}\boldsymbol{W}_2\boldsymbol{W}_1'\boldsymbol{\Gamma}\boldsymbol{W}_1 - \boldsymbol{\Gamma}\boldsymbol{W}_2\boldsymbol{W}_2'\boldsymbol{\Gamma}\boldsymbol{W}_1 + \boldsymbol{\Gamma}\boldsymbol{W}_2\boldsymbol{W}_2'\boldsymbol{\Gamma}\boldsymbol{W}_1 - \boldsymbol{\Gamma}\boldsymbol{W}_2\boldsymbol{W}_2'\boldsymbol{\Gamma}\boldsymbol{W}_2\|_F \\
\leq& \|\boldsymbol{\Gamma}(\boldsymbol{W}_1 - \boldsymbol{W}_2)\boldsymbol{W}_1'\boldsymbol{\Gamma}\boldsymbol{W}_1\|_F + \|\boldsymbol{\Gamma}\boldsymbol{W}_2(\boldsymbol{W}_1 - \boldsymbol{W}_2)'\boldsymbol{\Gamma}\boldsymbol{W}_1\|_F + \|\boldsymbol{\Gamma}\boldsymbol{W}_2\boldsymbol{W}_2'\boldsymbol{\Gamma}(\boldsymbol{W}_1 - \boldsymbol{W}_2)\|_F \\
\leq& \|\boldsymbol{\Gamma}\|_2^2(\|\boldsymbol{W}_1\|_2^2 + \|\boldsymbol{W}_1\|_2\|\boldsymbol{W}_2\|_2 + \|\boldsymbol{W}_2\|_2^2)\|\boldsymbol{W}_1 - \boldsymbol{W}_2\|_F \quad \text{(by Lemma 3)} \\
\leq& 3K\|\boldsymbol{\Gamma}\|_2^2\|\boldsymbol{W}_1 - \boldsymbol{W}_2\|_F
\end{aligned}
$$

Therefore,

$$
\|\nabla_{\boldsymbol{W}} H(\boldsymbol{W}_1, \boldsymbol{\Gamma}) - \nabla_{\boldsymbol{W}} H(\boldsymbol{W}_2, \boldsymbol{\Gamma})\|_F \leq 4(\|\boldsymbol{\Lambda}\|_2\|\boldsymbol{\Gamma}\|_2 + 3K\|\boldsymbol{\Gamma}\|_2^2)\|\boldsymbol{W}_1 - \boldsymbol{W}_2\|_F.
$$

For all $\boldsymbol{\Gamma}_1, \boldsymbol{\Gamma}_2 \in \mathbb{R}^{K\times K}$,

$$
\begin{aligned}
&\|\nabla_{\boldsymbol{\Gamma}} H(\boldsymbol{W}, \boldsymbol{\Gamma}_1) - \nabla_{\boldsymbol{\Gamma}} H(\boldsymbol{W}, \boldsymbol{\Gamma}_2)\|_F \\
=& \| - 2\boldsymbol{W}(\boldsymbol{\Lambda} - \boldsymbol{W}'\boldsymbol{\Gamma}_1\boldsymbol{W})\boldsymbol{W}' + 2\boldsymbol{W}(\boldsymbol{\Lambda} - \boldsymbol{W}'\boldsymbol{\Gamma}_2\boldsymbol{W})\boldsymbol{W}'\|_F \\
=& 2\|\boldsymbol{W}\boldsymbol{W}'(\boldsymbol{\Gamma}_1 - \boldsymbol{\Gamma}_2)\boldsymbol{W}\boldsymbol{W}'\|_F \\
\leq& 2\|\boldsymbol{W}\boldsymbol{W}'\|_2^2\|\boldsymbol{\Gamma}_1 - \boldsymbol{\Gamma}_2\|_F \quad \text{(by Lemma 3)} \\
\leq& 2\|\boldsymbol{W}\|_2^4\|\boldsymbol{\Gamma}_1 - \boldsymbol{\Gamma}_2\|_F. \quad \text{(by Lemma 1)}
\end{aligned}
$$

Proof of assumption (vi): Since $\boldsymbol{W}_t \in \mathcal{C}_{\boldsymbol{W}}$ and $\boldsymbol{\Gamma}_t \in \mathcal{C}_{\boldsymbol{\Gamma}}$ for all $t \in \mathbb{N}$, and $\mathcal{C}_{\boldsymbol{W}}$ and $\mathcal{C}_{\boldsymbol{\Gamma}}$ are compact sets, $L_1(\boldsymbol{\Gamma}) = 4(\|\boldsymbol{\Lambda}\|_2\|\boldsymbol{\Gamma}\|_2 + 3K\|\boldsymbol{\Gamma}\|_2^2)$ and $L_2(\boldsymbol{W}) = 2\|\boldsymbol{W}\|_2^4$ are bounded. By Lemma 4, $\|\boldsymbol{W}\|_2 \geq \frac{1}{\sqrt{S}}\|\boldsymbol{W}\|_1 = \frac{1}{\sqrt{S}}$ and $\|\boldsymbol{\Gamma}\|_2 \geq \frac{1}{\sqrt{S}}\|\boldsymbol{\Gamma}\|_1 \geq \frac{\epsilon}{\sqrt{S}}$. Therefore, $2\|\boldsymbol{W}_t\|_2^4 \geq \frac{2}{S^2}$ and $4(\|\boldsymbol{\Lambda}\|_2\|\boldsymbol{\Gamma}_t\|_2 + 3K\|\boldsymbol{\Gamma}_t\|_2^2) \geq 4(\epsilon\|\boldsymbol{\Lambda}\|_2/\sqrt{S} + 3K\epsilon^2/S)$ for all $t \in \mathbb{N}$.

F Simulating antecedent population coancestry through NORmal To Anything (NORTA)

NORmal To Anything (NORTA) algorithm. The NORmal To Anything (NORTA) algorithm is a transformation-based method for generating random vectors with given marginal distributions and a given covariance matrix. The goal of the NORTA method is to define a K-dimensional random vector $\boldsymbol{X}$ with the following properties:

(i) $X_u \sim F_u$, $u = 1, \ldots, K$, where $\{F_u\}$ are marginal cumulative distribution functions,

(ii) $\mathbb{C}(\boldsymbol{X}) = \boldsymbol{\Sigma}_{\boldsymbol{X}} = \{\sigma_{uv,\boldsymbol{X}}\}$.

The NORTA algorithm represents $\boldsymbol{X}$ as a transformation of a K-dimensional, multivariate Normal vector $\boldsymbol{Z} = (Z_1, Z_2, \ldots, Z_K)'$ with covariance matrix $\mathbb{C}(\boldsymbol{Z}) = \boldsymbol{\Sigma}_{\boldsymbol{Z}} = \{\sigma_{uv,\boldsymbol{Z}}\}$, where $\sigma_{uu,\boldsymbol{Z}} = 1$.

Alg D: NORTA algorithm

input : Marginal cumulative distribution functions F_u for $u = 1, 2, \ldots, K$, and the covariance matrix $\boldsymbol{\Sigma}_{\boldsymbol{X}}$

let $\boldsymbol{\Sigma}_{\boldsymbol{Z}} = \{\sigma_{uv,\boldsymbol{Z}}\}$ be a $K \times K$ identity matrix $\boldsymbol{I}$
for $u = 1, \ldots, K-1$ **do**
  for $v = u+1, \ldots, K$ **do**
    find σ^* such that $\mathbb{E}[X_u X_v] = \mathbb{E}[F_u^{-1}(\Phi(Z_u)) F_v^{-1}(\Phi(Z_v))]$
    let $\sigma_{uv,\boldsymbol{Z}} = \sigma_{vu,\boldsymbol{Z}} = \sigma^*$
simulate $\boldsymbol{Z}$ from the multivariate Normal distribution with mean-vector $\mathbf{0}$ and covariance matrix $\boldsymbol{\Sigma}_{\boldsymbol{Z}}$
let $\boldsymbol{X}$ be the transformation of $\boldsymbol{Z}$ where $X_u = F_u^{-1}(\Phi(Z_u))$ for $u = 1, \ldots, K$.
return $\boldsymbol{X}$

Φ is the univariate Normal$(0,1)$ cumulative distribution function (cdf); $F_u^{-1}(t) = \inf\{x : F_u(x) \geq t\}$ denotes the inverse cdf.

Note that $\mathbb{E}[X_u], \mathbb{E}[X_v]$, $\mathbb{V}(X_u)$ and $\mathbb{V}(X_v)$ are determined by F_u and F_v, implying $\mathbb{E}[X_u X_v]$ is determined by F_u and F_v. Let φ_σ denote a bivariate Normal probability density function with means 0, variances 1, and with the correlation (also the covariance in this case) σ between the two random variables. Then

$$\mathbb{E}[F_u^{-1}(\Phi(Z_u)) F_v^{-1}(\Phi(Z_v))] = \int_{-\infty}^{\infty} \int_{-\infty}^{\infty} F_{X_u}^{-1}(\Phi(z_u)) F_{X_v}^{-1}(\Phi(z_v)) \varphi_\sigma(z_u, z_v) dz_u dz_v. \quad (7)$$

Determining σ to yield the desired covariance is equivalent to solving the root of the function

$$\begin{aligned} g(\sigma) &= \mathbb{E}[F_u^{-1}(\Phi(Z_u))F_v^{-1}(\Phi(Z_v))] - \mathbb{E}[X_u X_v] \\ &= \int_{-\infty}^{\infty}\int_{-\infty}^{\infty} F_{X_u}^{-1}(\Phi(z_u))F_{X_v}^{-1}(\Phi(z_v))\varphi_\sigma(z_u, z_v)dz_u dz_v - \mathbb{E}[X_u X_v]. \end{aligned}$$

We will denote this root by σ^* where $g(\sigma^*) = 0$.

Applying NORTA to generate coancestry among antecedent populations. We applied the NORTA algorithm with $\boldsymbol{X} = \boldsymbol{p}_i$, $F_u = \text{BN}(a_i, \lambda_{uu})$, and $\sigma_{uv,\boldsymbol{X}} \equiv a_i(1-a_i)\lambda_{uv}$ for $i \in 1, \ldots, m$. In this scenario,

$$\mathbb{E}[X_u X_v] = a_i^2 + a_i(1-a_i)\lambda_{uv}$$

We note that there is no closed form solution for σ^*. We adopted the Newton-Raphson method to perform a numerical search for σ^*. Let

$$g(\sigma; a_i, \lambda_{uv}) = \mathbb{E}[F_u^{-1}(\Phi(Z_u))F_v^{-1}(\Phi(Z_v))] - a_i^2 - a_i(1-a_i)\lambda_{uv}.$$

It follows that

$$g'(\sigma; a_i, \lambda_{uv}, F_u, F_v) = \mathbb{E}\left[F_u^{-1}(\Phi(Z_u))F_v^{-1}(\Phi(Z_v))\left(\frac{\sigma}{1-\sigma^2} + \frac{z_u z_v - \sigma(z_u^2 - \sigma z_u z_v + z_v^2)}{(1-\sigma^2)^2}\right)\right].$$

The Newton iteration for finding σ^* is then given by

$$\sigma \leftarrow \sigma - \frac{g(\sigma; a_i, \lambda_{uv}, F_u, F_v)}{g'(\sigma; a_i, \lambda_{uv}, F_u, F_v)}.$$

We calculated $g(\sigma; a_i, \lambda_{uv}, F_u, F_v)$ and $g'(\sigma; a_i, \lambda_{uv}, F_u, F_v)$ via numeric integration, leading to Alg E for simulating antecedent population allele frequencies with the desired coancestry.

Alg E: NORTA algorithm for simulating $\boldsymbol{P}$

input: Ancestral allele frequencies $\boldsymbol{a}$ and coancestry among antecedent populations $\boldsymbol{\Lambda}$

for $i = 1, \ldots, m$ **do**
  let $\boldsymbol{\Sigma_Z}$ be an $K \times K$ identity matrix $\boldsymbol{I}$
  for $u = 1, \ldots, K - 1$ **do**
    for $v = u + 1, \ldots, K$ **do**
      let $\sigma = \lambda_{uv} / \sqrt{\lambda_{uu}\lambda_{vv}}$
      while *not converged* **do**
        let $\sigma \leftarrow \sigma - \frac{g(\sigma; a_i, \lambda_{uv}, F_u, F_v)}{g'(\sigma; a_i, \lambda_{uv}, F_u, F_v)}$
      let $\sigma_{uv,\boldsymbol{Z}} = \sigma_{vu,\boldsymbol{Z}} = \sigma$
  generate $\boldsymbol{z} \sim \mathcal{N}(\boldsymbol{0}, \boldsymbol{\Sigma_Z})$
  let $\boldsymbol{p}_i$ be a K-dimensional vector where $p_{iu} = F_u^{-1}(\Phi(z_u))$ for $u = 1, \ldots, K$
return $\boldsymbol{P}$

Φ is the univariate Normal$(0, 1)$ cumulative distribution function (cdf); $F_u = \text{BN}(a_i, \lambda_{uu})$; $F_u^{-1}(t) = \inf\{x : F_u(x) \geq t\}$ denotes the inverse cdf of F_u.

G Constructing a dendrogram from population coancestry

To construct a dendrogram from a matrix of population-level coancestry $\boldsymbol{\Lambda}$, we calculate a distance matrix $\boldsymbol{D}$ from $\boldsymbol{\Lambda}$ according to:

$$d_{uv} = \begin{cases} 0 & \text{if } u = v \\ \max(\lambda_{uv}) - \lambda_{uv} & \text{if } u \neq v. \end{cases}$$

We then apply the standard agglomerative clustering method to $\boldsymbol{D}$ using "weighted pair group method with arithmetic mean" (WPGMA) to obtain a dendrogram.

SUPPLEMENTARY SIMULATIONS

H Generating $\mathbf{\Lambda}$

To simulate $\mathbf{\Lambda}$ under the super admixture model, we simulated a $K \times K$ matrix $\boldsymbol{A}$ with elements drawn independently from Uniform$(0, 0.3)$. We then set $\mathbf{\Lambda} = \boldsymbol{A}'\boldsymbol{A}$. To simulate $\mathbf{\Lambda}$ under the standard admixture model, we let $\mathbf{\Lambda}$ be a diagonal matrix whose diagonal elements are generated independently from Uniform$(0, 1)$. For both scenarios, we varied $K = 3, 6, 9$ and sampled 100 instances of $\mathbf{\Lambda}$ for each K. Our simulation resulted in 300 instances of $\mathbf{\Lambda}$ under the standard admixture model and 300 instances of $\mathbf{\Lambda}$ under the super admixture model.

I Generating $\boldsymbol{Q}$

We adopted the spatial model from [2] to generate admixture proportions reflecting real data. This model represents the admixture process as a diffusion on a one-dimensional geography. It assumes K independent populations equally spaced at positions $x_0, x_0 + 1, \ldots, x_0 + K - 1$ on an infinite line. If all populations begin to diffuse at time $t = 0$ at the same diffusion rate, then population u will be distributed as a Gaussian with mean $\mu_u = x_0 + u - 1$ and standard deviation σ. Therefore, under the spatial model an individual j sampled at the position j will have the admixture proportions shown as follows:

$$\boldsymbol{q}_j = (q_{1j}, q_{2j}, \ldots, q_{Kj})' = \left(\frac{\mathcal{N}(j;\mu_1,\sigma^2)}{\sum_{u=1}^{K} \mathcal{N}(j;\mu_u,\sigma^2)}, \quad \frac{\mathcal{N}(j;\mu_2,\sigma^2)}{\sum_{u=1}^{K} \mathcal{N}(j;\mu_u,\sigma^2)}, \quad \ldots, \quad \frac{\mathcal{N}(j;\mu_K,\sigma^2)}{\sum_{u=1}^{K} \mathcal{N}(j;\mu_u,\sigma^2)} \right)$$

where $\mathcal{N}(;\mu, \sigma^2)$ denotes a Normal(μ, σ^2) distribution. We chose $\sigma^2 = 0.5$ and $n = 2000$ in our simulations.

J Evaluating algorithms for estimating coancestry among antecedent populations

To evaluate Alg 1 and Alg B, we generated 300 unique combinations of $(\mathbf{\Lambda}, \boldsymbol{Q})$ under the standard admixture model and 300 unique combinations of $(\mathbf{\Lambda}, \boldsymbol{Q})$ under the super admixture model. For each pair of $(\mathbf{\Lambda}, \boldsymbol{Q})$, we calculated the corresponding $\mathbf{\Theta} = \boldsymbol{Q}'\mathbf{\Lambda}\boldsymbol{Q}$. If the super admixture model is assumed, we applied Alg 1 with a random initial matrix $\mathbf{\Lambda}_0$ to estimate $\mathbf{\Lambda}$.

If the standard admixture model is assumed, we applied Alg B with a random initial matrix $\mathbf{\Lambda}_0$ to estimate $\mathbf{\Lambda}$. We recorded values of $\mathbf{\Lambda}_t$ per iteration. We quantified the differences between $\mathbf{\Lambda}$ and $\mathbf{\Lambda}_t$ by $\frac{\|\mathbf{\Lambda}_t - \mathbf{\Lambda}\|_F}{\|\mathbf{\Lambda}\|_F}$, and visualized the change over iterations in Fig A. We validated that both algorithms are capable of generating a sequence of $\mathbf{\Lambda}_t$ such that the difference between $\mathbf{\Lambda}$ and $\mathbf{\Lambda}_t$ decreases as $t \to \infty$.

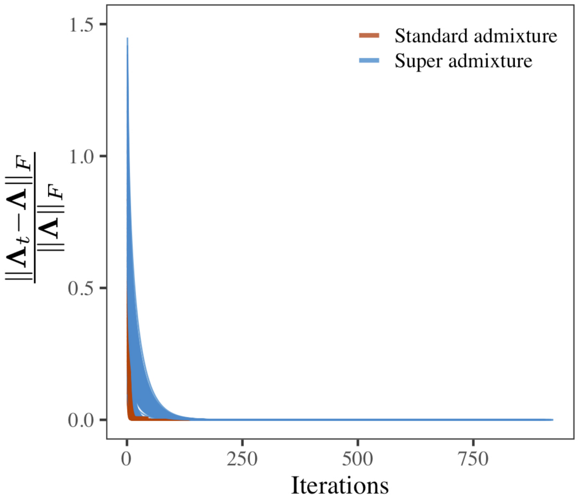

Figure A: **Convergence of Alg 1 and Alg B measured by relative Frobenius error, $\frac{\|\mathbf{\Lambda}_t - \mathbf{\Lambda}\|_F}{\|\mathbf{\Lambda}\|_F}$, across iterations.** The blue lines show $\mathbf{\Lambda}_t$ calculated from the Alg 1 when the true $\mathbf{\Lambda}$ is simulated from the super admixture model. The red lines show $\mathbf{\Lambda}_t$ calculated from the Alg B when the true $\mathbf{\Lambda}$ is simulated from the standard admixture model.

K Evaluating algorithms for generating antecedent population allele frequencies

To evaluate Alg 4 and Alg E,, we assessed whether they could generate allele frequencies that satisfy the moments of the super admixture model:

$$\mathbb{E}[p_{iu}|T] = a_i$$
$$\mathbb{V}[p_{iu}|T] = a_i(1 - a_i)\lambda_{uu}$$
$$\mathbb{C}[p_{iu}, p_{iv}|T] = a_i(1 - a_i)\lambda_{uv}$$

To achieve this, we generated 300 unique combinations of $(a, \mathbf{\Lambda})$ where a is a scalar and is simulated from Uniform$(0, 1)$; $\mathbf{\Lambda}$ assumes the super admixture model and is simulated as previously described. For each pair of $(a, \mathbf{\Lambda})$, we generated $B = 100,000$ replications

of the m-vector allele frequencies $\boldsymbol{p}^{(b)}$ from the double-admixture method (Alg 4) or from the NORTA method (Alg E). Then we calculated the empirical mean and the empirical covariance matrix as $\hat{\boldsymbol{a}} = \frac{1}{B}\sum_{b=1}^{B} \boldsymbol{p}^{(b)}$ and $\hat{\boldsymbol{C}} = \frac{1}{B}\sum_{b=1}^{B}(\boldsymbol{p}^{(b)} - \hat{\boldsymbol{a}})(\boldsymbol{p}^{(b)} - \hat{\boldsymbol{a}})\prime$, respectively. We measured the differences between empirical moments and the desired moments by $\|\hat{\boldsymbol{a}} - \boldsymbol{a}\|_2/\|\boldsymbol{a}\|_2$ and $\|\hat{\boldsymbol{C}} - \boldsymbol{C}\|_F/\|\boldsymbol{C}\|_F$, where $\boldsymbol{a}$ denotes a $K \times 1$ dimensional vector whose entries are all equal to a and $\boldsymbol{C} = a(1-a)\boldsymbol{\Lambda}$. We found that $\|\hat{\boldsymbol{a}} - \boldsymbol{a}\|_2/\|\boldsymbol{a}\|_2$ is generally less than 0.02 and $\|\hat{\boldsymbol{C}} - \boldsymbol{C}\|_F/\|\boldsymbol{C}\|_F$ is generally less than 0.04 for both algorithms (Fig B). These findings confirmed the performance of both algorithms.

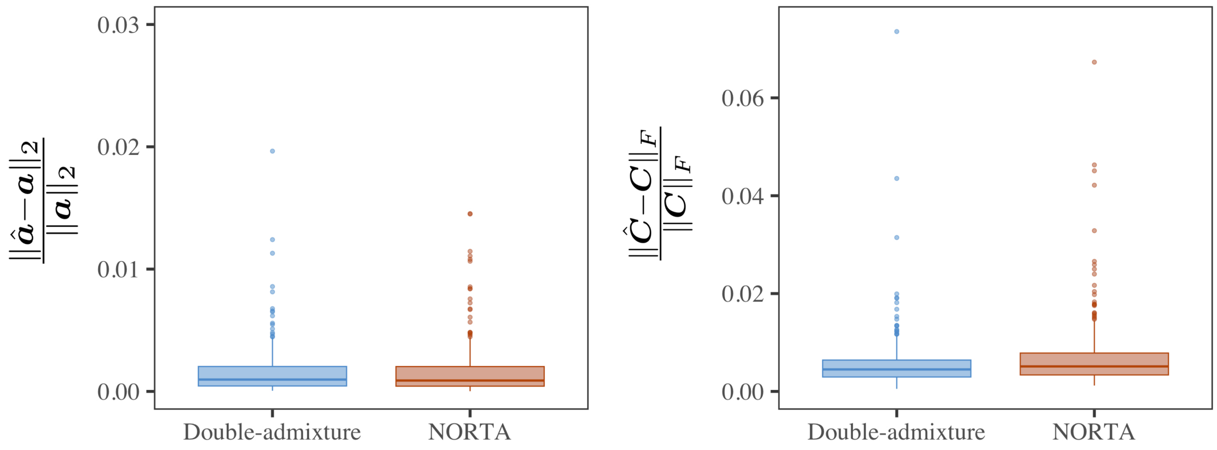

Figure B: **Validation of Alg 4 (double-admixture) and Alg E (NORTA).** The left panel shows the relative Frobenius norm error for ancestral allele frequencies, $\frac{\|\hat{\boldsymbol{a}}-\boldsymbol{a}\|_2}{\|\boldsymbol{a}\|_2}$, across 300 simulations. The right panel shows the relative Frobenius norm error for population-level coancestry, $\frac{\|\hat{\boldsymbol{C}}-\boldsymbol{C}\|_F}{\|\boldsymbol{C}\|_F}$, across 300 simulations. Different colors indicate results from different algorithms.

L Evaluating the algorithm for generating genotypes from the super admixture model

To evaluate Alg 5, we assessed whether this algorithm is capable of generating genotypes that satisfy the moment constraints imposed by the super admixture model. More specifically, we examined if the estimated individual-level coancestry agrees with $\boldsymbol{Q}'\boldsymbol{\Lambda}\boldsymbol{Q}$ and if the estimated coancestry among antecedent populations agrees with $\boldsymbol{\Lambda}$.

To check these, we generated 300 unique combinations of $(\boldsymbol{\Lambda}, \boldsymbol{Q})$ under the super admixture model as previously described. For each pair of $(\boldsymbol{\Lambda}, \boldsymbol{Q})$, we (i) simulated ancestral allele frequencies $\boldsymbol{a}$ ($m = 500,000$) by generating each a_i independently from Uniform$(0, 1)$,

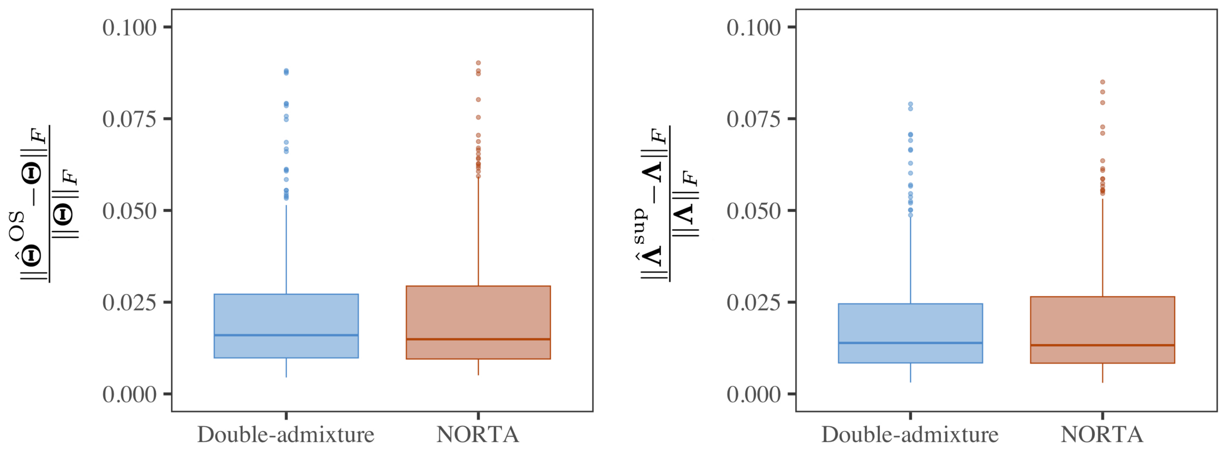

Figure C: **Validation of Alg 5.** The left panel shows the relative Frobenius norm errors for individual-level coancestry, $\|\hat{\mathbf{\Theta}}^{\mathrm{OS}} - \mathbf{\Theta}\|_F/\|\mathbf{\Theta}\|_F$, across 300 simulations. The right panel shows the relative Frobenius norm error for population-level coancestry $\|\hat{\mathbf{\Lambda}}^{\mathrm{sup}} - \mathbf{\Lambda}\|_F/\|\mathbf{\Lambda}\|_F$ across 300 simulations. The color indicates the algorithm used to simulate antecedent population allele frequencies.

(ii) generated genotypes $\boldsymbol{X}$ using Alg 5, (iii) estimated the individual-level coancestry $\hat{\mathbf{\Theta}}^{\mathrm{OS}}$ and (iv) applied Alg 1 to estimate $\hat{\mathbf{\Lambda}}^{\mathrm{sup}}$ with $\hat{\mathbf{\Theta}}^{\mathrm{OS}}$ and $\boldsymbol{Q}$ as inputs. In (ii), a matrix of antecedent population allele frequencies $\boldsymbol{P}$ is generated at an intermediate step. We used both the double-admixture method and the NORTA method for generating $\boldsymbol{P}$ to compare their performances. For (iii), the OS estimate utilizes the minimum pairwise coancestry equal to 0, which might not hold here. We used the strategy described in Appendix C to adapt the OS estimate to our simulation. We assessed the agreement between $\hat{\mathbf{\Theta}}^{\mathrm{OS}}$ and $\mathbf{\Theta}$ and between $\hat{\mathbf{\Lambda}}^{\mathrm{sup}}$ and $\mathbf{\Lambda}$ by $\|\hat{\mathbf{\Theta}}^{\mathrm{OS}} - \mathbf{\Theta}\|_F/\|\mathbf{\Theta}\|_F$ and $\|\hat{\mathbf{\Lambda}}^{\mathrm{sup}} - \mathbf{\Lambda}\|_F/\|\mathbf{\Lambda}\|_F$, respectively. In Fig C, we observed the majority of $\|\hat{\mathbf{\Theta}}^{\mathrm{OS}} - \mathbf{\Theta}\|_F/\|\mathbf{\Theta}\|_F$ and $\|\hat{\mathbf{\Lambda}}^{\mathrm{sup}} - \mathbf{\Lambda}\|_F/\|\mathbf{\Lambda}\|_F$ are less than 0.05 regardless of the method used for simulating antecedent population allele frequencies. These observations demonstrated our simulated genotypes satisfied the desired moment constraints.

M Null p-value distribution of the hypothesis test of standard admixture versus super admixture

Recall the hypothesis test of the standard admixture model (null) versus the super admixture model (alternative):

$$H_0 : \max\left(\{\lambda_{uv}\}_{u\neq v}\right) = 0 \text{ (standard admixture model)}$$
$$H_1 : \max\left(\{\lambda_{uv}\}_{u\neq v}\right) > 0 \text{ (super admixture model)}$$

To check whether the true null hypothesis p-values calculated by Alg 6 are stochastically greater than or equal to the Uniform$(0, 1)$ distribution, we generated 300 unique combinations of $(\boldsymbol{\Lambda}, \boldsymbol{Q})$ under the standard admixture model. For each pair of $(\boldsymbol{\Lambda}, \boldsymbol{Q})$, we (i) simulated ancestral allele frequencies $\boldsymbol{a}$ ($m = 500, 000$) by generating each a_i independently from Uniform$(0, 1)$, (ii) generated genotypes $\boldsymbol{X}$ from the standard admixture model, (iii) applied Alg 6 to compute the p-values. We compared the empirical distribution of the p-values against Uniform$(0, 1)$. Fig D shows that our proposed method is conservative, meaning it has a maximum type I error probability less than or equal to the nominal level of the test.

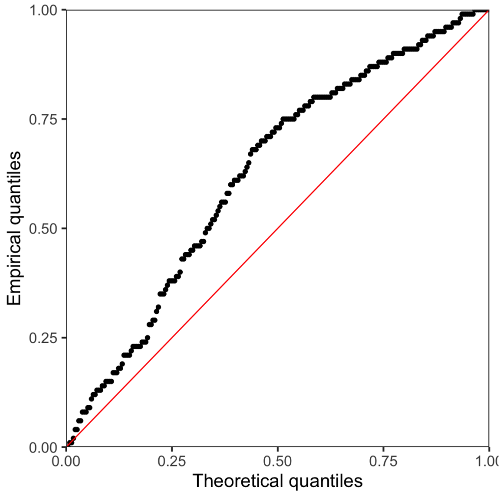

Figure D: **Quantile–quantile plot comparing null p-values from Alg 6 with the Uniform(0, 1) distribution.**

SUPPLEMENTARY ANALYSES OF HUMAN STUDIES

N Data processing

HO data. The Human Origins data sets from refs. [5] and [6] were obtained from:

(i) Human Origins present-day individuals analyzed in ref. [5] are available at: `https://reich.hms.harvard.edu/sites/reich.hms.harvard.edu/files/inline-files/NearEastPublic.tar.gz`

(ii) Human Origins present-day individuals analyzed in ref. [6] available at: `https://reich.hms.harvard.edu/sites/reich.hms.harvard.edu/files/inline-files/SkoglundEtAl2016_Pacific_FullyPublic(3).tar.gz`

The downloaded data sets are in the Eigensoft package format. We converted them to the PLINK format with the Eigensoft `convertf` function. We then merged the main Human Origins data set with the Pacific data set. These data sets have non-overlapping individuals that were genotyped using the same microarray platform. We excluded individuals from singleton subpopulations and the ancient individuals from the Lapita-Vanuatu population. We excluded SNPs with minor allele frequency (MAF) less than 0.01. The final data set has $486,981$ SNPs and 2124 individuals.

HGDP data. The Human Genome Diversity Project (HGDP) data from ref. [7] are available at: `https://ngs.sanger.ac.uk/production/hgdp/hgdp_wgs.20190516/`. Here, we started with the assembled version of HGDP, provided by the PLINK [8]. PLINK hosts the assembled data at the following links:

(i) .pgen: `https://www.dropbox.com/s/hppj1g1gzygcocq/hgdp_all.pgen.zst?dl=1`

(ii) .pvar: `https://www.dropbox.com/s/1mmkq0bd9ax8rng/hgdp_all.pvar.zst?dl=1`

(iii) .psam: `https://www.dropbox.com/s/0zg57558fqpj3w1/hgdp.psam?dl=1`

The associated annotations can be found at the FTP site: `ftp://ngs.sanger.ac.uk/production/hgdp/hgdp_wgs.20190516/metadata/hgdp_wgs.20190516.metadata.txt`. We downloaded and unzipped the assembled HGDP data from the links listed above. We preserved loci that (i) are autosomal, biallelic SNPs, (ii) have MAF ≥ 0.01 and (iii) are in

approximate linkage equilibrium with each other (`PLINK --indep-pairwise 1000kb 0.3`). The final data set has $997,431$ SNPs and 929 individuals.

TGP data. The raw TGP phase 3 data are available at `https://ftp.1000genomes.ebi.ac.uk/vol1/ftp/data_collections/1000G_2504_high_coverage/working/20220422_3202_phased_SNV_INDEL_SV/`, from ref. [9]. Here, we started with the assembled version of TGP, provided by the PLINK. PLINK hosts the assembled data at the following links:

(i) `.pgen`: `https://www.dropbox.com/s/j72j6uciq5zuzii/all_hg38.pgen.zst?dl=1`

(ii) `.pvar`: `https://www.dropbox.com/s/vx09262b4k1kszy/all_hg38.pvar.zst?dl=1`

(iii) `.psam`: `https://www.dropbox.com/s/2e87z6nc4qexjjm/hg38_corrected.psam?dl=1`

The associated annotations can be found from the following links.

(i) Pedigree information: `http://ftp.1000genomes.ebi.ac.uk/vol1/ftp/data_collections/1000G_2504_high_coverage/working/1kGP.3202_samples.pedigree_info.txt`

(ii) Population information: `http://ftp.1000genomes.ebi.ac.uk/vol1/ftp/data_collections/1000G_2504_high_coverage/20130606_g1k_3202_samples_ped_population.txt`.

We downloaded and unzipped the assembled TGP data from the links listed above. We preserved loci that (i) are autosomal, biallelic SNPs, (ii) are variants in the Yoruba individuals, (iii) have MAF ≥ 0.05 and (iv) are in approximate linkage equilibrium with each other (`PLINK --indep-pairwise 1000kb 0.3`). The final data set has $712,998$ SNPs and 2583 individuals.

AMR subset of TGP. We identified individuals in the TGP data set marked as `AMR` to create the AMR subset of TGP. We preserved loci that (i) are autosomal, biallelic SNPs, (ii) are variants in the Yoruba individuals, (iii) have MAF ≥ 0.01 and (iv) are in approximate linkage equilibrium with each other (`PLINK --indep-pairwise 1000kb 0.3`). The final data set has $555,145$ SNPs and 353 individuals.

IND data. We obtained the Indian data set from the authors of ref. [10]. We merged this data set with the Central/South Asia population and the East Asia population of HGDP. These data sets have non-overlapping individuals that were genotyped using the same microarray platform. We excluded SNPs with MAF < 0.01 and SNPs with MAF differences greater than 0.2 to resolve the allele flipping issue. We also excluded SNPs with missing rates in IND or in the HGDP subset greater than 0.005. We applied this filter to keep high quality variants. The final data set has $221,499$ SNPs and 698 individuals.

O HGDP study analysis

We observed good concordance between the HGDP individual-level coancestry estimated using OS and super admixture (Fig E), which is consistent with our knowledge of early human migration [11–14]. We noted that the earliest major split occurred between Africa and MiddleEast from an out-of-Africa migration around 50 to 60 kya, resulting in the divergence between Sub-Saharan Africans and the remaining human populations. Another major split occurred between Central / South Asia and East Asia, revealing the separation between West Eurasians and East Asians around 40 to 45 kya. Among the East Asia clade, the Oceanians have the highest within subpopulation coancestry and lowest between subpopulation coancestry, consistent with the theory that Oceanians split earliest from the remaining East Asians.

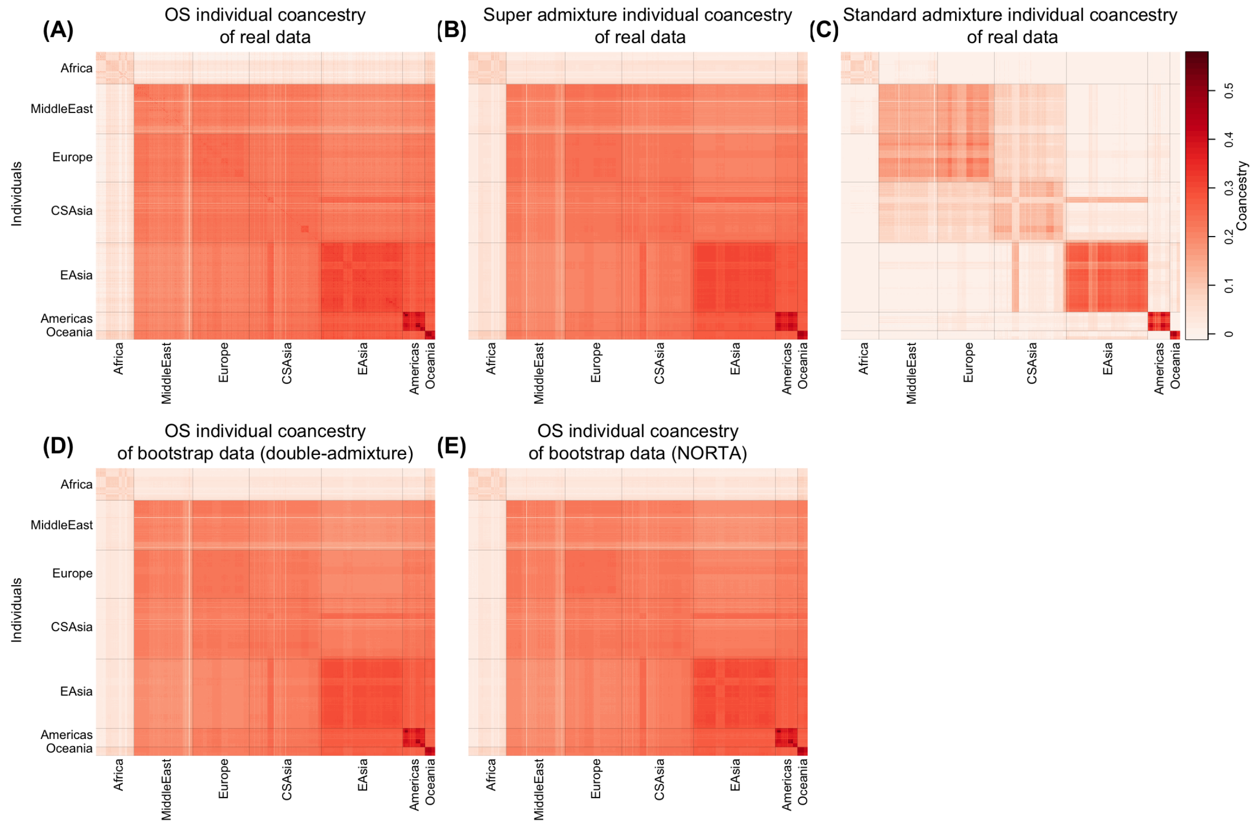

Figure E: **Heatmaps of individual-level coancestry estimates in HGDP.** Each cell represents the estimated coancestry between a pair of individuals, with warmer colors indicating higher values. **(A)**–**(C)** show estimates from the observed genotypes using the Ochoa–Storey (OS) method, the super admixture method, and the standard admixture method, respectively. **(D)** shows estimates from bootstrap re-sampled genotypes using the OS method, with antecedent allele frequencies simulated under a double-admixture approach. **(E)** shows estimates from bootstrapped re-sampled genotypes using the OS method, with antecedent allele frequencies simulated using the NORTA approach.

We confirmed that the super admixture antecedent population coancestry estimates are also compatible with known early human dispersals (Fig F). Specifically, in Fig FB the deepest split occurred roughly between individuals from Sub-Saharan Africa represented by the antecedent populations S_1 and S_2 and individuals outside of Sub-Saharan Africa represented by the remaining antecedent populations. Individuals outside of Sub-Saharan Africa further branched into two lineages: the West Eurasians represented by antecedent populations S_3 and S_4, and the East Asians represented by antecedent populations S_5, S_6, and S_7. The Oceanians represented by S_7 split from the majority of ancestral East Asians, while the remaining East Asians further diverged into present-day Asians (S_5) and present-day Americans (S_6).

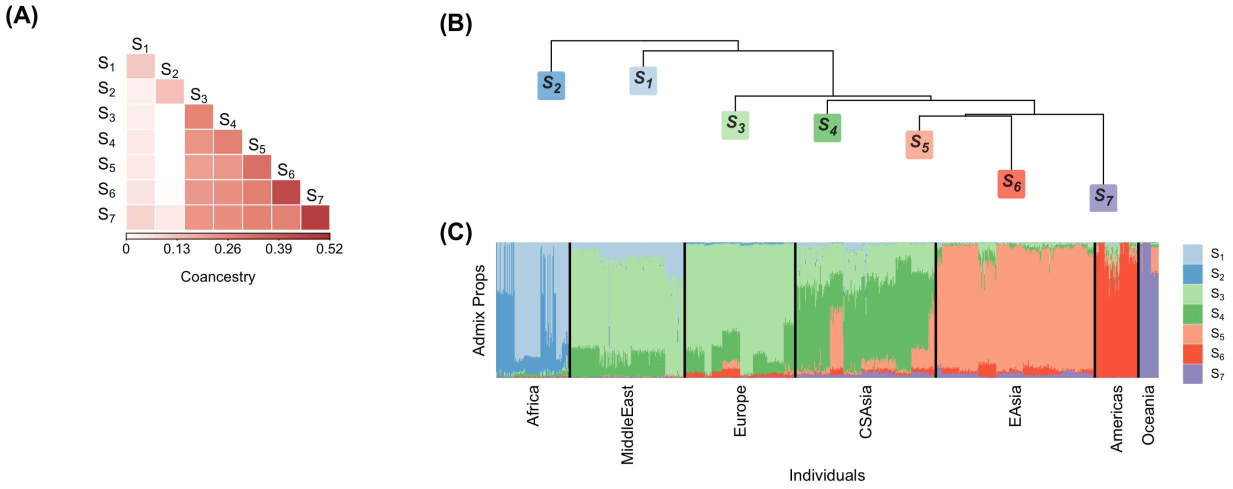

Figure F: **Visualization of population-level coancestry and admixture proportions in the HGDP. (A)** Heatmap of antecedent population coancestry estimates. **(B)** Dendrogram representation of the antecedent population coancestry estimates. **(C)** Stacked bar plot of admixture proportions.

P TGP study analysis

We also observed good concordance between the TGP individual-level coancestry estimated using OS and super admixture (Fig G), which is consistent with our knowledge of early human migration [11–14]. Similarly to our analysis of HO and HGDP, the earliest major split is between AFR and the other populations, which reflects the divergence between Sub-Saharan Africans and the remaining of human populations. Another split occurs between EUR and EAS, revealing the separation between West Eurasians and East Asians.

As in our analysis of HO and HGDP, there is agreement between the estimated antecedent population coancestry and existing results. In Fig HB, the deepest split occurred roughly between individuals from Sub-Saharan Africa represented by antecedent population S_1 and individuals outside of Sub-Saharan Africa represented by the rest of the antecedent populations. We also noted the divergence between the Europeans represented by antecedent population S_3, and the Asians represented by antecedent populations S_2, S_4 and S_5. The Americans sampled in TGP appear to have a higher European ancestry compared to that in the HO and HGDP data sets.

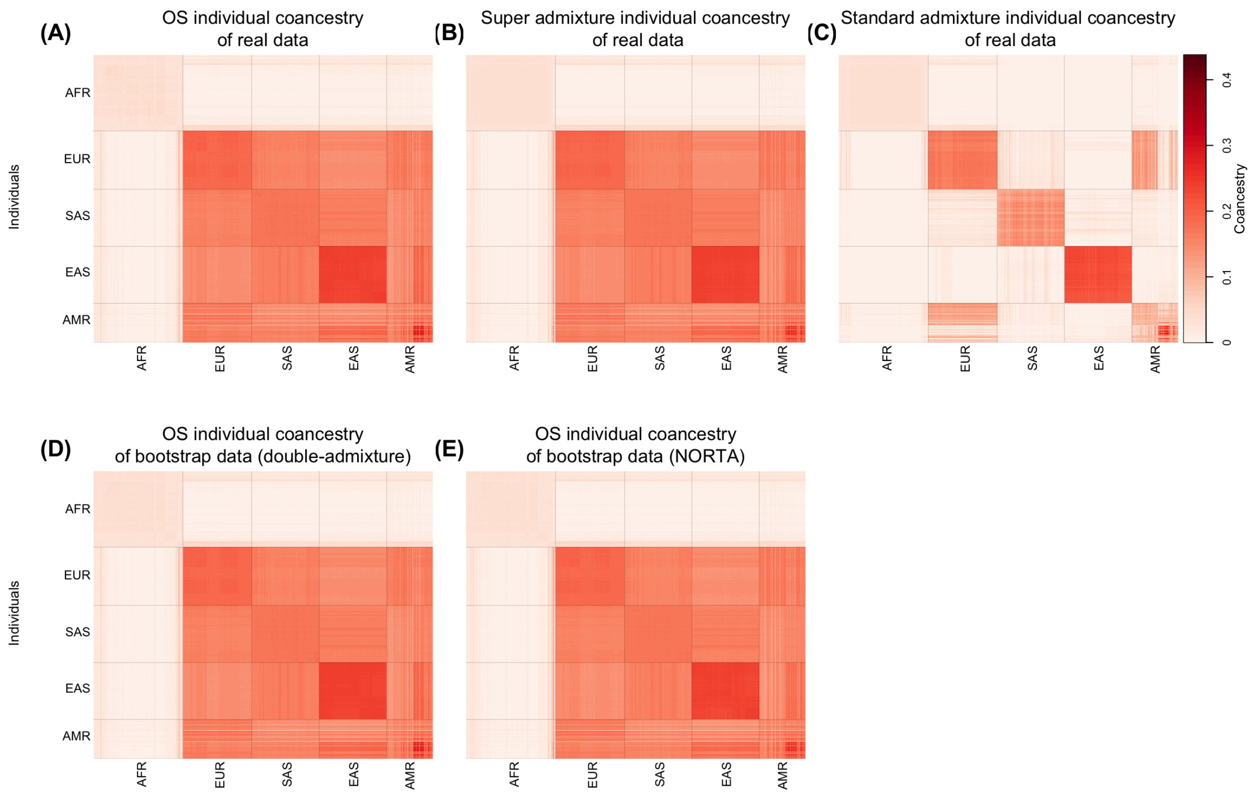

Figure G: **Heatmaps of individual-level coancestry estimates in TGP.** Each cell represents the estimated coancestry between a pair of individuals, with warmer colors indicating higher values. **(A)**–**(C)** show estimates from the observed genotypes using the Ochoa–Storey (OS) method, the super admixture method, and the standard admixture method, respectively. **(D)** shows estimates from bootstrap re-sampled genotypes using the OS method, with antecedent allele frequencies simulated under a double-admixture approach. **(E)** shows estimates from bootstrapped re-sampled genotypes using the OS method, with antecedent allele frequencies simulated using the NORTA approach.

Q Confirming significant hypothesis tests of standard admixture versus super admixture in the human studies

We applied Alg 6 to each of the five human data sets to statistically evaluate the presence of coancestry among antecedent populations. Each panel of Fig I shows the distribution of the $B = 1000$ bootstrap null test-statistics for each data set. The observed test-statistic U_{obs} for each data set is noted on the top-right of each panel. In each data set U_{obs} exceeded all

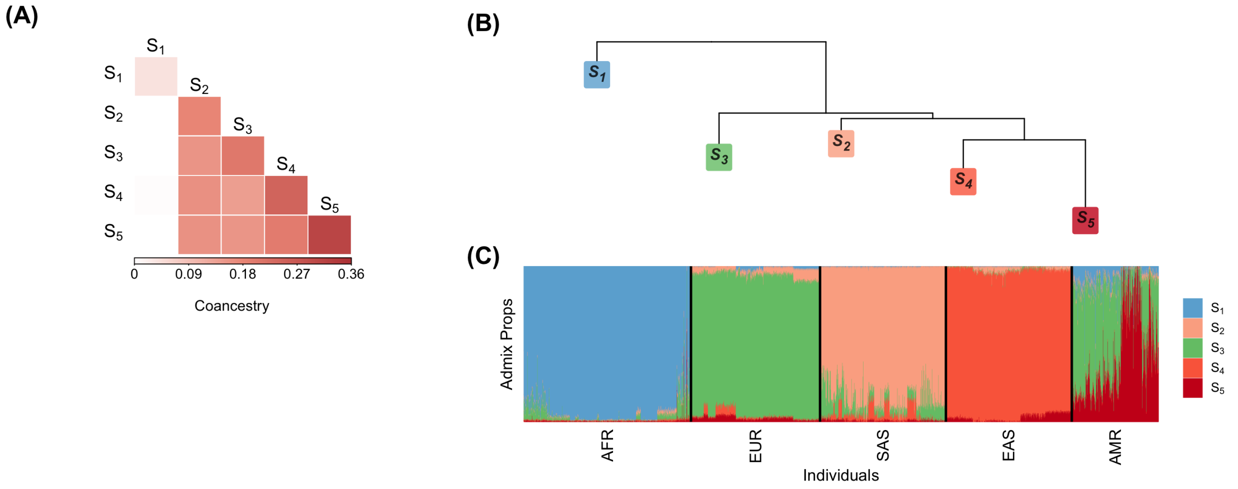

Figure H: **Visualization of population-level coancestry and admixture proportions in the TGP. (A)** Heatmap of antecedent population coancestry estimates. **(B)** Dendrogram representation of the antecedent population coancestry estimates. **(C)** Stacked bar plot of admixture proportions.

bootstrap null test-statistics, implying p-value < 0.001 for each.

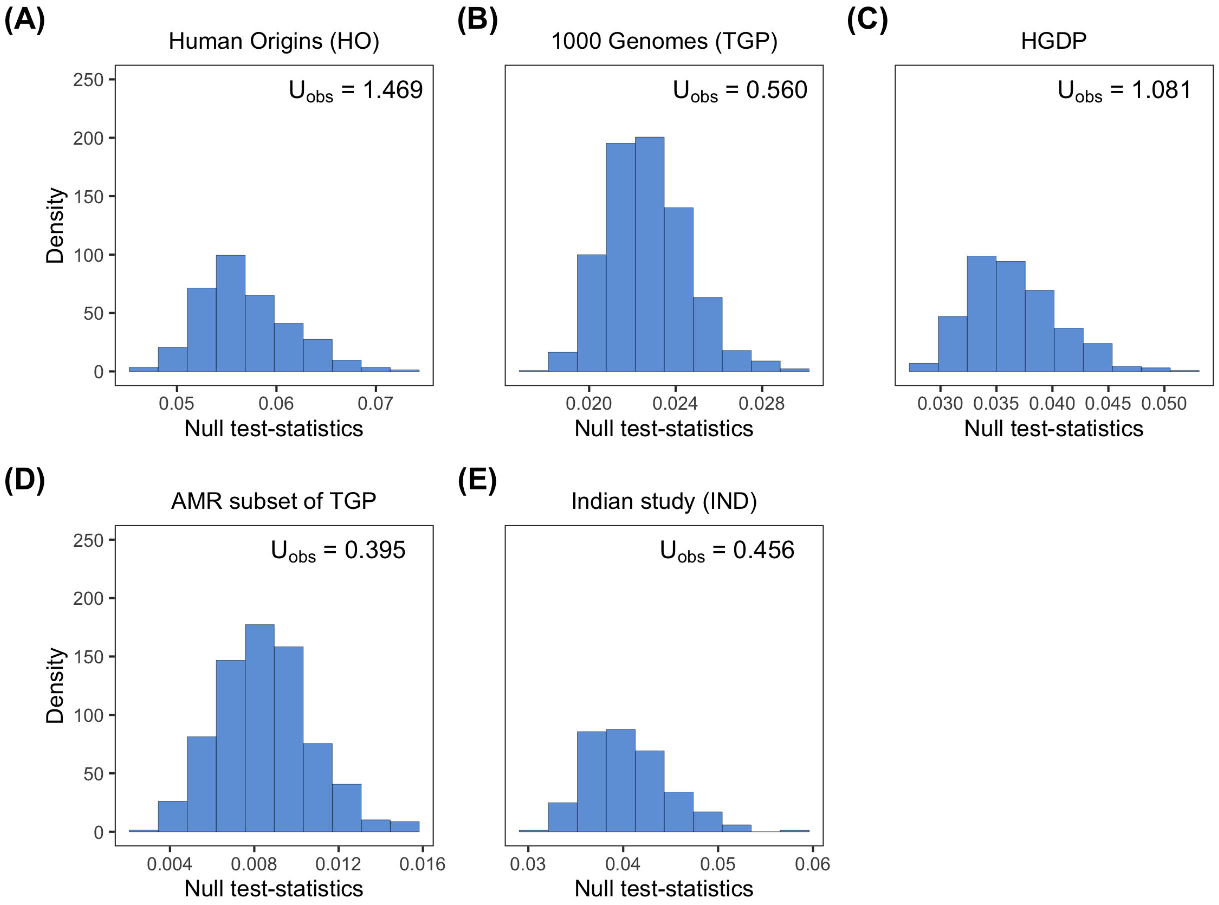

Figure I: **Distributions of null test-statistics for the hypothesis tests of standard admixture versus super admixture.** The observed test-statistic U_{obs} for each data set is noted on the top-right of each panel.

R Comparing the individual-level coancestry estimates

In Table A, we compared $\hat{\mathbf{\Theta}}^{\text{sup}}$ and $\hat{\mathbf{\Theta}}^{\text{std}}$ to the general OS estimate of individual-level coancestry $\hat{\mathbf{\Theta}}^{\text{OS}}$ from ref. [2] on the five data sets. The super admixture coancestry estimate has about 10 to 40 times smaller distance to the OS estimate compared to the standard admixture estimate.

	$\frac{1}{n}\|\hat{\mathbf{\Theta}}^{\text{sup}} - \hat{\mathbf{\Theta}}^{\text{OS}}\|_F$	$\frac{1}{n}\|\hat{\mathbf{\Theta}}^{\text{std}} - \hat{\mathbf{\Theta}}^{\text{OS}}\|_F$
HO	0.003	0.114
AMR	0.006	0.124
IND	0.006	0.052
HGDP	0.008	0.146
TGP	0.002	0.087

	$\|\hat{\mathbf{\Theta}}^{\text{sup}} - \hat{\mathbf{\Theta}}^{\text{OS}}\|_F / \|\hat{\mathbf{\Theta}}^{\text{OS}}\|_F$	$\|\hat{\mathbf{\Theta}}^{\text{std}} - \hat{\mathbf{\Theta}}^{\text{OS}}\|_F / \|\hat{\mathbf{\Theta}}^{\text{OS}}\|_F$
HO	0.021	0.751
AMR	0.023	0.507
IND	0.073	0.613
HGDP	0.040	0.724
TGP	0.017	0.700

Table A: The relative Frobenius norm differences between $\hat{\mathbf{\Theta}}^{\text{OS}}$ and $\hat{\mathbf{\Theta}}^{\text{sup}}$, and between $\hat{\mathbf{\Theta}}^{\text{OS}}$ and $\hat{\mathbf{\Theta}}^{\text{std}}$ across the five data sets. $\hat{\mathbf{\Theta}}^{\text{OS}}$, $\hat{\mathbf{\Theta}}^{\text{sup}}$, and $\hat{\mathbf{\Theta}}^{\text{std}}$ denote the individual-level coancestry estimated by the Ochoa–Storey (OS) method, the super admixture method, and the standard admixture method, respectively.

S Selecting the number of antecedent populations

We utilized the structural Hardy-Weinberg (sHWE) framework [15] for determining the number of antecedent populations K, as outlined in that work. The approach considers a range of K values for a model of structure that results in estimated IAFs, which is the case for our framework. For each K, a hypothesis test is performed for each SNP of the assumption that $x_{ij}|\pi_{ij} \sim \text{Binomial}(2, \pi_{ij})$ based on the estimates $\hat{\pi}_{ij}$ and a goodness-of-fit statistic with a parametric bootstrap null distribution, for $i = 1, \dots, m$ and $j = 1, \dots, n$. This results in m p-values per value of K.

As proposed in the sHWE framework, for each value of K, we (i) calculated the m sHWE p-values, (ii) binned the sHWE p-values into equal-sized bins (number of bins, $C = 150$), (iii) removed the first bin $[0, 1/C)$, and (iv) calculated the following negative entropy that measures how well the sHWE p-values follow the Uniform$(0, 1)$ distribution,

$$\sum_{c=2}^{C} f_c \log_{10} f_c,$$

where f_c denotes the proportion of p-values in the c-th bin.

We determined K to be the value achieving the minimum negative entropy. In the event

of a plateau where the entropy is more or less the same over a range of K, we chose a small value of K, where the plateau began. This resulted in $K = 11$ for HO, $K = 7$ for HGDP, $K = 5$ for TGP, $K = 7$ for IND, and $K = 3$ for AMR.

In Fig J, we observed a consistent decline in $\|\hat{\mathbf{\Theta}}^{\text{sup}} - \hat{\mathbf{\Theta}}^{\text{OS}}\|_F / \|\hat{\mathbf{\Theta}}^{\text{OS}}\|_F$ as K increased. In Fig K, we noted an increase in $\|\hat{\mathbf{\Theta}}^{\text{std}} - \hat{\mathbf{\Theta}}^{\text{OS}}\|_F / \|\hat{\mathbf{\Theta}}^{\text{OS}}\|_F$ as K increased. This implies the standard admixture fit cannot be improved with a larger K. In the following two subsections, we show that the results for HO and IND are similar over a range of K close to the values that we selected.

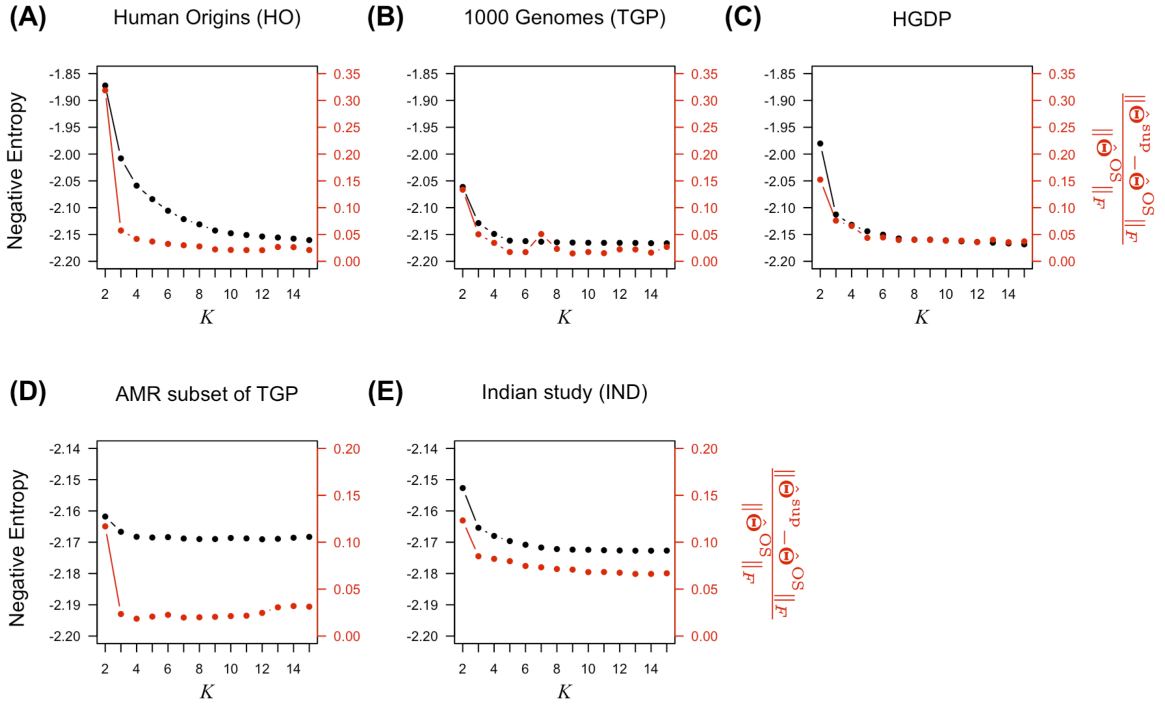

Figure J: The negative entropy, $\sum_{c=2}^{C} f_c \log_{10} f_c$, and the relative Frobenius norm error from the super admixture model, $\frac{\|\hat{\mathbf{\Theta}}^{\text{sup}} - \hat{\mathbf{\Theta}}^{\text{OS}}\|_F}{\|\hat{\mathbf{\Theta}}^{\text{OS}}\|_F}$, evaluated across different numbers of antecedent populations.

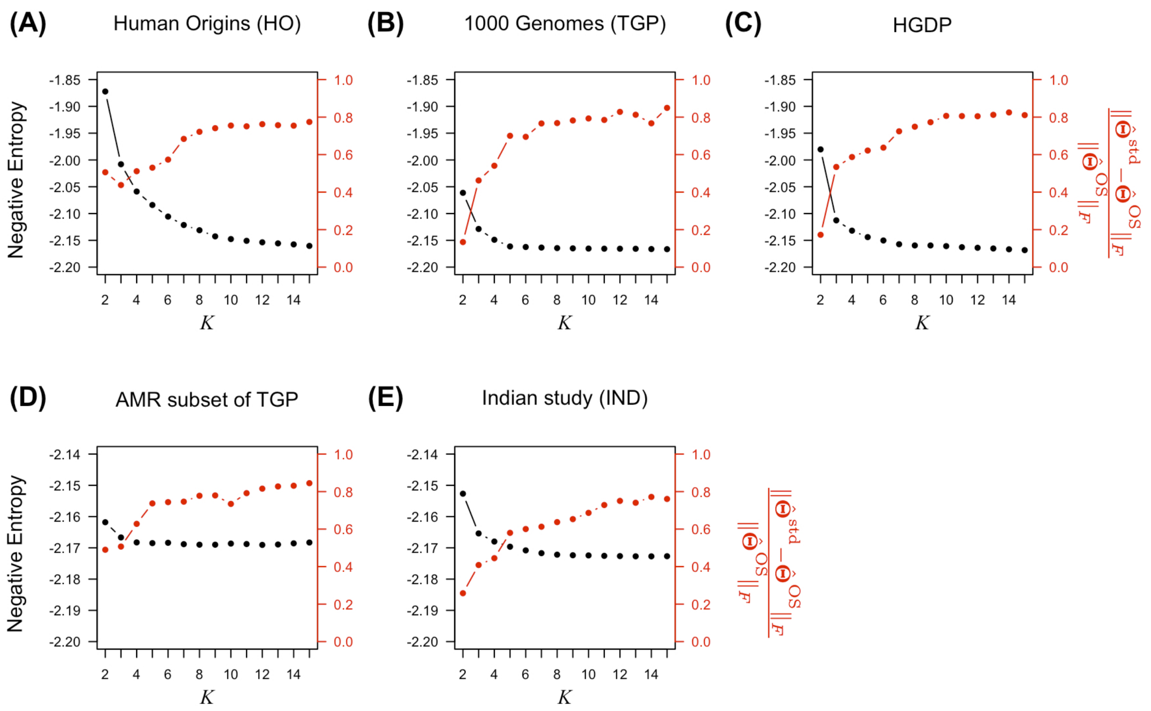

Figure K: The negative entropy, $\sum_{c=2}^{C} f_c \log_{10} f_c$, and the relative Frobenius norm error from the standard admixture model, $\frac{\|\hat{\boldsymbol{\Theta}}^{\text{std}} - \hat{\boldsymbol{\Theta}}^{\text{OS}}\|_F}{\|\hat{\boldsymbol{\Theta}}^{\text{OS}}\|_F}$, evaluated across different numbers of antecedent populations.

T Analysis of HO over a range of antecedent population numbers

In our analysis of HO, we utilized $K = 11$ antecedent populations. We also analyzed the HO data set for $K = 7, 8, 9, 10$ in this section to demonstrate the choice of K did not have a major impact on our results and conclusions. In Figs L to O, we observed the estimated antecedent population coancestries and the admixture proportions were consistent for $K = 7, 8, 9, 10$.

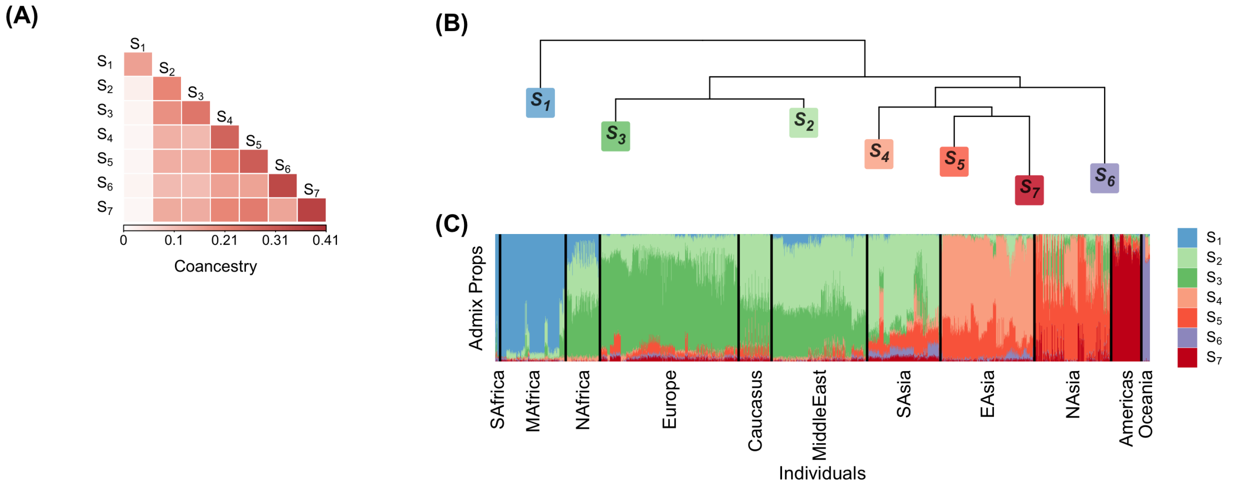

Figure L: (A) Heatmap of antecedent population coancestry estimates in HO with $K = 7$. (B) Dendrogram representation of the antecedent population coancestry estimates. (C) Stacked bar plot of admixture proportions.

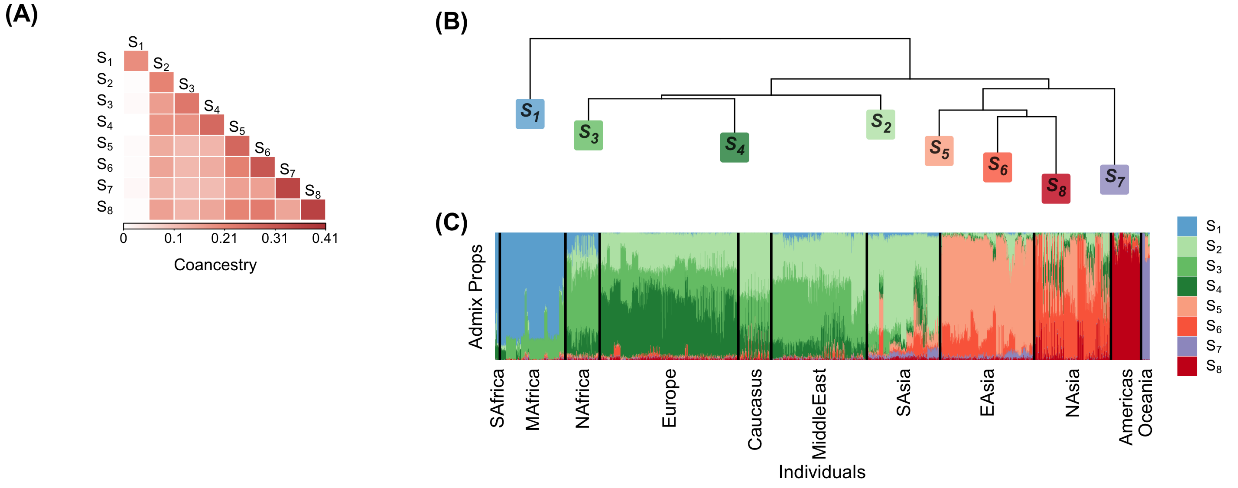

Figure M: (A) Heatmap of antecedent population coancestry estimates in HO with $K = 8$. (B) Dendrogram representation of the antecedent population coancestry estimates. (C) Stacked bar plot of admixture proportions.

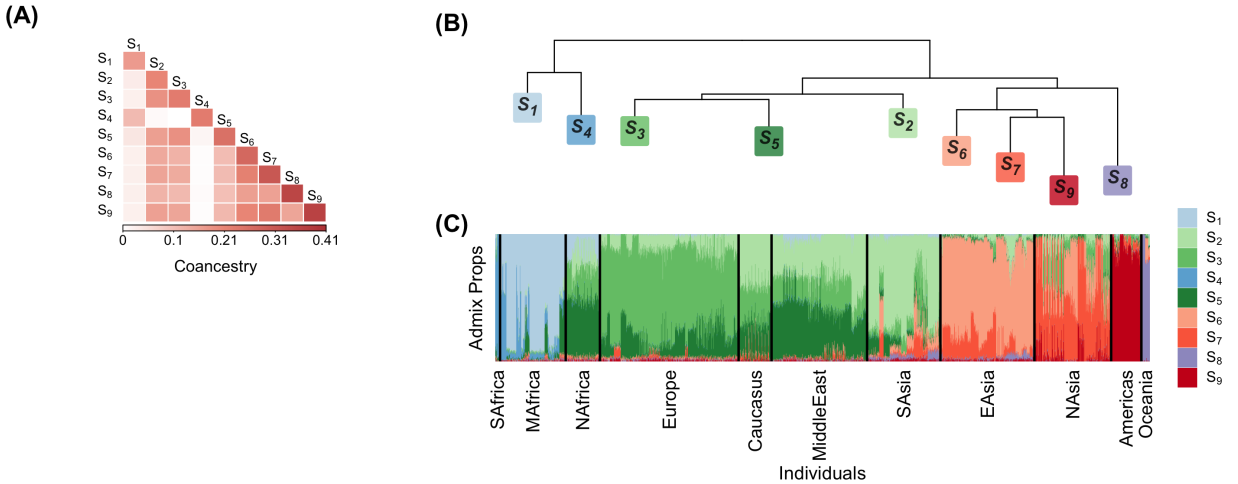

Figure N: (A) Heatmap of antecedent population coancestry estimates in HO with $K = 9$. (B) Dendrogram representation of the antecedent population coancestry estimates. (C) Stacked bar plot of admixture proportions.

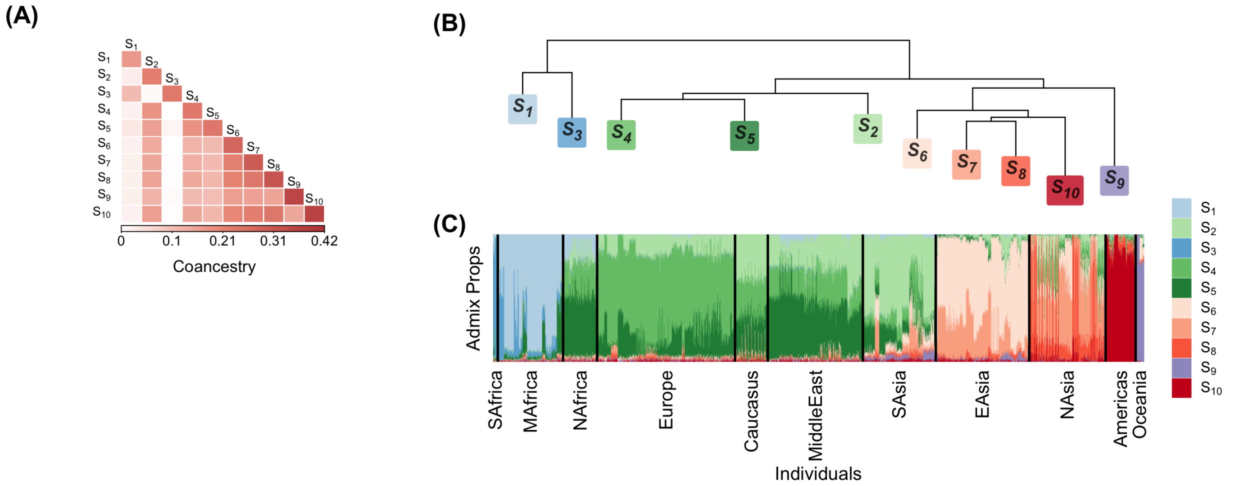

Figure O: (A) Heatmap of antecedent population coancestry estimates in HO with $K = 10$. (B) Dendrogram representation of the antecedent population coancestry estimates. (C) Stacked bar plot of admixture proportions.

U Analysis of IND over a range of antecedent population numbers

In our analysis of IND, we utilized $K = 7$ antecedent populations. In Figs P to S, we observe the estimated antecedent population coancestries and the admixture proportions were consistent for $K = 3, 4, 5, 6$.

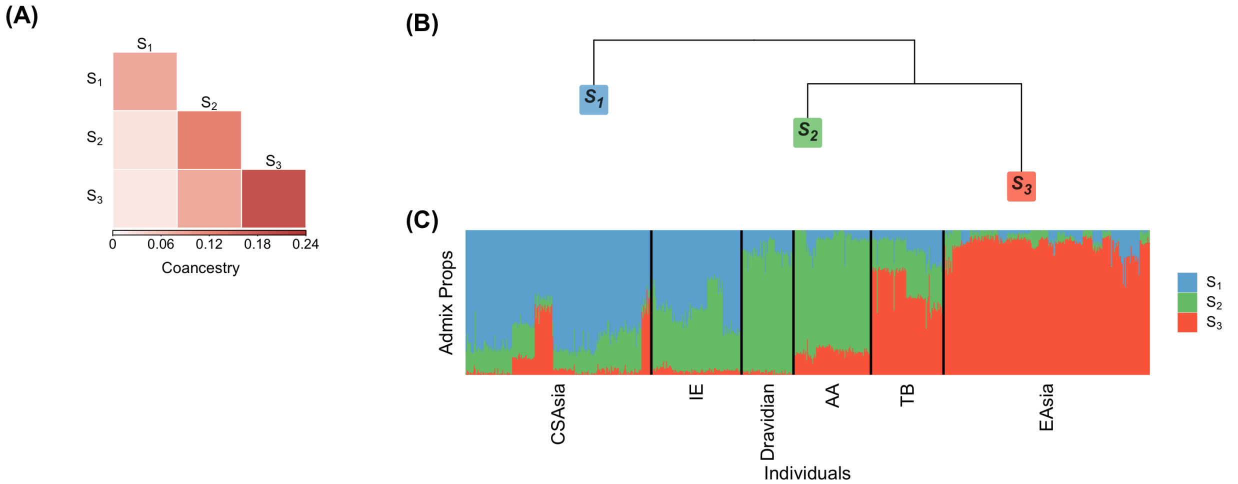

Figure P: (A) Heatmap of antecedent population coancestry estimates in the merged data sets of IND with Central/South Asians and East Asians of HGDP with $K = 3$. (B) Dendrogram representation of the antecedent population coancestry estimates. (C) Stacked bar plot of admixture proportions.

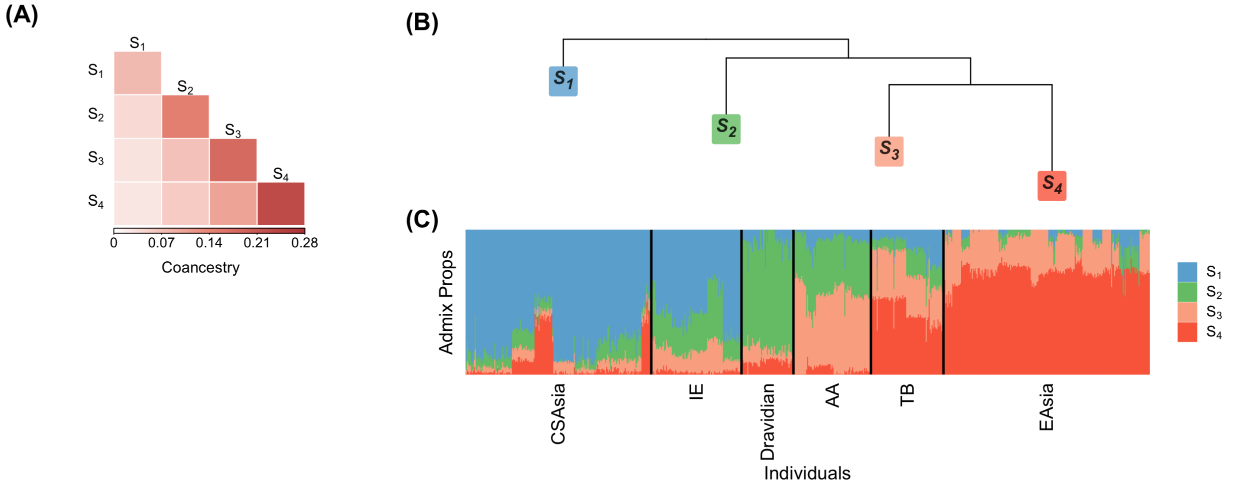

Figure Q: (A) Heatmap of antecedent population coancestry estimates in the merged data sets of IND with Central/South Asians and East Asians of HGDP with $K = 4$. (B) Dendrogram representation of the antecedent population coancestry estimates. (C) Stacked bar plot of admixture proportions.

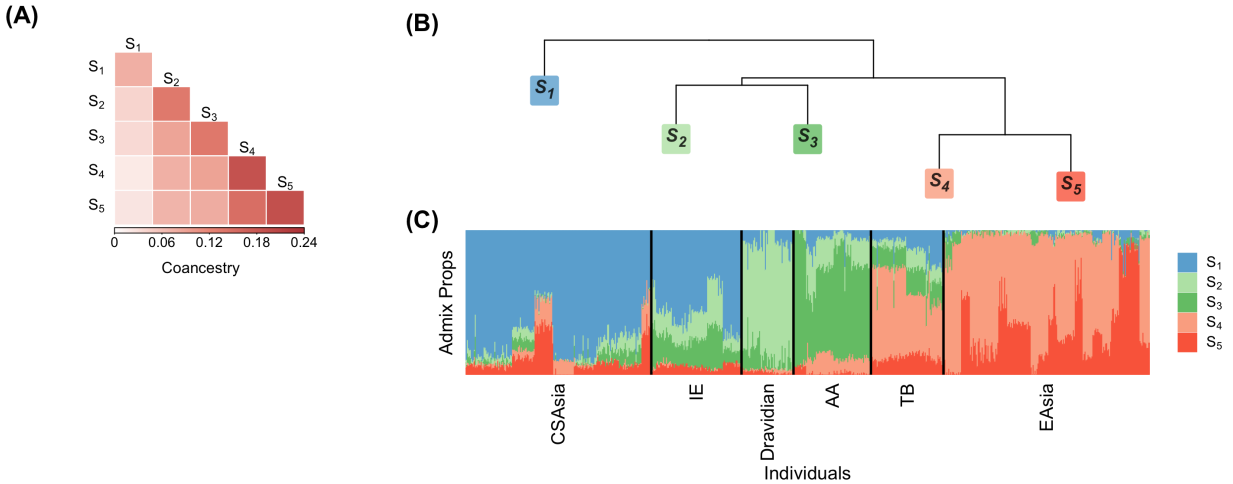

Figure R: (A) Heatmap of antecedent population coancestry estimates in the merged data sets of IND with Central/South Asians and East Asians of HGDP with $K = 5$. (B) Dendrogram representation of the antecedent population coancestry estimates. (C) Stacked bar plot of admixture proportions.

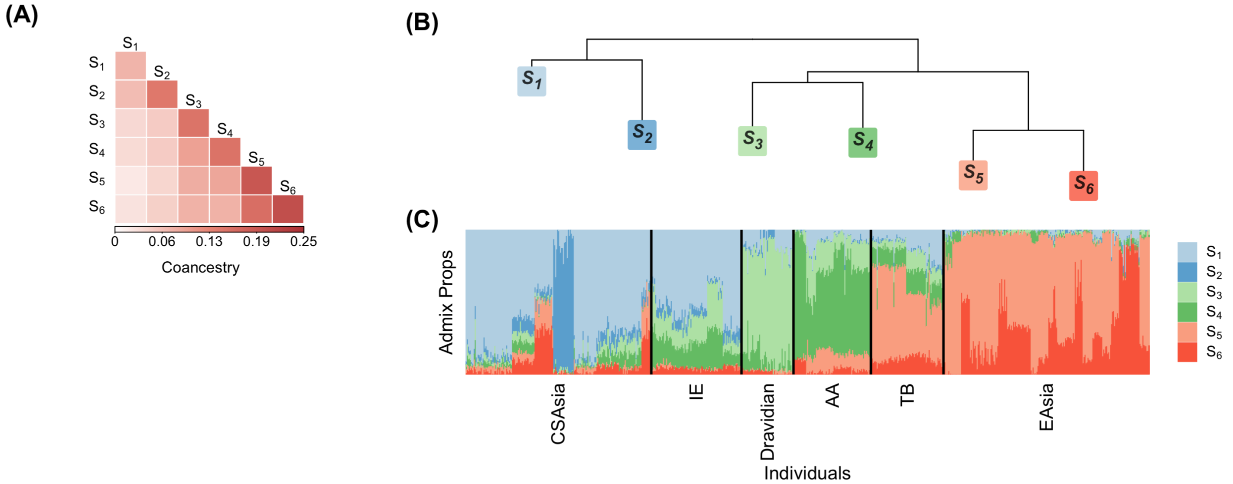

Figure S: (A) Heatmap of antecedent population coancestry estimates in the merged data sets of IND with Central/South Asians and East Asians of HGDP with $K = 6$. (B) Dendrogram representation of the antecedent population coancestry estimates. (C) Stacked bar plot of admixture proportions.

References

[1] J. Bolte, S. Sabach, and M. Teboulle. “Proximal alternating linearized minimization for nonconvex and nonsmooth problems”. *Mathematical Programming* 146(1) (2014), pp. 459–494.

[2] A. Ochoa and J. D. Storey. “Estimating FST and kinship for arbitrary population structures”. *PLoS Genetics* 17(1) (2021), e1009241.

[3] A. Ochoa and J. D. Storey. *popkin: Estimate Kinship and FST under Arbitrary Population Structure*. R package version 1.3.23. 2023.

[4] P. L. Combettes and V. R. Wajs. “Signal recovery by proximal forward-backward splitting”. *Multiscale Modeling & Simulation* 4(4) (2005), pp. 1168–1200.

[5] I. Lazaridis et al. “Genomic insights into the origin of farming in the ancient Near East”. *Nature* 536(7617) (2016), pp. 419–424.

[6] P. Skoglund et al. “Genomic insights into the peopling of the Southwest Pacific”. *Nature* 538(7626) (2016), pp. 510–513.

[7] A. Bergström et al. “Insights into human genetic variation and population history from 929 diverse genomes”. *Science* 367(6484) (2020), eaay5012.

[8] S. Purcell et al. “PLINK: A Tool Set for Whole-Genome Association and Population-Based Linkage Analyses”. *American Journal of Human Genetics* 81(3) (2007), pp. 559–575.

[9] M. Byrska-Bishop et al. “High-coverage whole-genome sequencing of the expanded 1000 Genomes Project cohort including 602 trios”. *Cell* 185(18) (2022), pp. 3426–3440.

[10] A. Basu, N. Sarkar-Roy, and P. P. Majumder. “Genomic reconstruction of the history of extant populations of India reveals five distinct ancestral components and a complex structure”. *Proceedings of the National Academy of Sciences* 113(6) (2016), pp. 1594–1599.

[11] J. D. Wall. “Inferring human demographic histories of non-African populations from patterns of allele sharing”. *The American Journal of Human Genetics* 100(5) (2017), pp. 766–772.

[12] M. Lipson and D. Reich. “A working model of the deep relationships of diverse modern human genetic lineages outside of Africa”. *Molecular Biology and Evolution* 34(4) (2017), pp. 889–902.

[13] R. Nielsen et al. “Tracing the peopling of the world through genomics”. *Nature* 541(7637) (2017), pp. 302–310.

[14] A. Bergström et al. “Origins of modern human ancestry”. *Nature* 590(7845) (2021), pp. 229–237.

[15] W. Hao and J. D. Storey. “Extending tests of Hardy–Weinberg equilibrium to structured populations”. *Genetics* 213(3) (2019), pp. 759–770.
